# *Plagl1* and *Lrrc58* control mammalian body size by triggering target-directed microRNA degradation of miR-322 and miR-503

**DOI:** 10.1101/gad.353138.125

**Published:** 2026-02-01

**Authors:** Collette A. LaVigne, Jaeil Han, He Zhang, Sihoon Cho, Minseon Kim, Komal Sethia, Bret M. Evers, Asha Acharya, Tsung-Cheng Chang, Joshua T. Mendell

**Affiliations:** 1 Department of Molecular Biology, University of Texas Southwestern Medical Center, Dallas, Texas 75390, USA;; 2 Department of Microbiology and Molecular Biology, Chungnam National University, Daejeon 34134, Republic of Korea;; 3 Department of Health Data Science and Biostatistics, Peter O'Donnell Jr. School of Public Health, University of Texas Southwestern Medical Center, Dallas, Texas 75390, USA;; 4 Department of Microbiology, University of Texas Southwestern Medical Center, Dallas, Texas 75390, USA;; 5 Department of Pathology, University of Texas Southwestern Medical Center, Dallas, Texas 75390, USA;; 6 Department of Ophthalmology, University of Texas Southwestern Medical Center, Dallas, Texas 75390, USA;; 7 Howard Hughes Medical Institute, University of Texas Southwestern Medical Center, Dallas, Texas 75390, USA;; 8 Harold C. Simmons Comprehensive Cancer Center, University of Texas Southwestern Medical Center, Dallas, Texas 75390, USA;; 9 Hamon Center for Regenerative Science and Medicine, University of Texas Southwestern Medical Center, Dallas, Texas 75390, USA

**Keywords:** embryonic growth, *Lrrc58*, *Plagl1*, TDMD, target-directed microRNA degradation, miR-322, miR-503, microRNA

## Abstract

Precise control of microRNA (miRNA) expression is critical during development. An important mechanism of miRNA regulation is target-directed microRNA degradation (TDMD), a pathway in which the binding of miRNAs to specialized trigger RNAs induces ubiquitylation and decay of associated argonaute (AGO) proteins by the ZSWIM8 ubiquitin ligase. Concomitant release of miRNAs results in their rapid turnover. ZSWIM8-deficient mice exhibit reduced body size, cardiopulmonary and neurodevelopmental defects, and perinatal lethality. Despite widespread dysregulation of miRNAs in these animals, the vast majority of presumptive trigger RNAs that induce decay of ZSWIM8-regulated miRNAs remain undefined. Here, using AGO cross-linking and sequencing of hybrids (AGO-CLASH), a high-throughput method for identifying miRNA binding sites, we report the identification of *Plagl1* as a TDMD trigger for miR-322-5p and *Lrrc58* and *Malat1* as TDMD triggers for miR-503-5p in mouse embryonic fibroblasts (MEFs). In mice, deletion of the miR-322-5p and miR-503-5p trigger sites in the *Plagl1* and *Lrrc58* 3′ UTRs, respectively, abrogated TDMD of these miRNAs and resulted in miR-322/503-dependent embryonic growth restriction, recapitulating a key feature of the *Zswim8*^−*/*−^ phenotype. Thus, *Plagl1* and *Lrrc58* act as triggers for degradation of miR-322-5p and miR-503-5p, revealing a noncoding function for these mRNAs as regulators of mammalian body size.

## Abstract (precis)

In this study, LaVigne et al. identify *Plagl1*, *Lrrc58*, and *Malat1* as trigger RNAs for the ZSWIM8-mediated, target-directed microRNA degradation (TDMD) of miR-322-5p and miR-503-p, respectively. The work highlights how such noncoding functions of these mRNAs, as well as the TDMD pathway, not only control miRNA turnover but also serve as regulators of mammalian growth.

MicroRNAs (miRNAs) are small noncoding RNAs that function as important post-transcriptional regulators of gene expression across eukaryotes (Bartel 2018). miRNAs are produced by a highly regulated biogenesis pathway that processes long primary miRNA transcripts (pri-miRNAs) into short ∼22 nt double-stranded RNA duplexes (Kim et al. 2025). One strand of the duplex, representing the mature miRNA, is loaded into an argonaute (AGO) protein, while the other strand, referred to as the passenger strand or miRNA*, is degraded. miRNA-loaded AGO proteins surveil mRNA and noncoding RNA transcripts for sites with complementarity to nucleotides 2–7 of the miRNA, termed the seed sequence. Base-pairing between the miRNA and target RNA enables AGO to recruit deadenylation and decapping factors, ultimately leading to repression of translation and/or accelerated degradation of the target (Jonas and Izaurralde 2015; Shang et al. 2023). The majority of mammalian genes have at least one conserved miRNA binding site (Friedman et al. 2009), allowing miRNAs to broadly sculpt gene expression during development and disease (Mendell and Olson 2012; Alberti and Cochella 2017; DeVeale et al. 2021). Consequently, elaborate mechanisms that control miRNA transcription, processing, and degradation have evolved in order to precisely control the timing and magnitude of gene silencing by this pathway (Winter et al. 2009; Gebert and MacRae 2019; Hiers et al. 2024).

The loading of a miRNA into an AGO protein shields it from degradation, usually conferring a long half-life that enables many cycles of target RNA repression (van Rooij et al. 2007; Gatfield et al. 2009). However, analyses of miRNA decay rates revealed a set of exceptional miRNAs that exhibit accelerated turnover (Hwang et al. 2007; Bail et al. 2010; Krol et al. 2010; Gantier et al. 2011; Rissland et al. 2011; Guo et al. 2015; Marzi et al. 2016; Kingston and Bartel 2019; Reichholf et al. 2019). It is now appreciated that a mechanism called target-directed miRNA degradation (TDMD) accounts for the enhanced decay of many of these short-lived miRNAs. TDMD is activated upon base-pairing of a miRNA to a specialized mRNA or noncoding RNA, referred to as a trigger RNA (Ameres et al. 2010; Cazalla et al. 2010). Our understanding of the base-pairing architecture and potentially other features that differentiate a canonical miRNA target site from a TDMD trigger site is still evolving. The majority of known trigger RNAs in *Drosophila* and mammals engage in extensive base-pairing with both the seed region and 3′ end of the miRNA (Bitetti et al. 2018; Ghini et al. 2018; Kleaveland et al. 2018; Li et al. 2021; Kingston et al. 2022; Sheng et al. 2023). However, a functional TDMD site with limited 3′ pairing was recently identified in *Drosophila* (Hiers et al. 2025), while in *Caenorhabditis elegans*, base-pairing of the miRNA seed is sufficient to initiate TDMD in a subset of cases (Donnelly et al. 2022; Stubna et al. 2025). TDMD requires a cullin–RING ubiquitin ligase complex containing the adapter protein ZSWIM8 (Han et al. 2020; Shi et al. 2020). Upon binding of a miRNA to a trigger site, the ZSWIM8 complex is believed to ubiquitylate the associated AGO protein, leading to proteasome-mediated degradation and concomitant release and decay of the miRNA. Extensive base-pairing between the miRNA and trigger, involving both the seed and miRNA 3′ end, results in a unique AGO conformation (Sheu-Gruttadauria et al. 2019) that is thought to be specifically recognized by ZSWIM8. How ZSWIM8 orthologs are activated in cases where accelerated miRNA degradation requires limited 3′ pairing remains a mystery (Donnelly et al. 2022; Hiers et al. 2025).

Identification of the protein machinery that mediates TDMD, and the deep conservation of ZSWIM8, has allowed investigation of the physiologic role and scope of miRNA regulation by this pathway in diverse metazoan species. In *C. elegans*, loss of the ZSWIM8 ortholog EBAX-1 causes defects in locomotion, egg-laying, male mating behaviors, and axon guidance (Wang et al. 2013). Small RNA sequencing documented >20 miRNAs that are stabilized in EBAX-1-deficient worms (Donnelly et al. 2022; Stubna et al. 2025). Dora, the *Drosophila* ortholog of ZSWIM8, is essential, with most *dora* knockout embryos failing to hatch into L1 larvae (Kingston et al. 2022; Molina-Pelayo et al. 2022). More than 20 Dora-regulated miRNAs have been identified in S2 cells and embryos (Shi et al. 2020; Kingston et al. 2022). Importantly, embryonic lethality in flies is partially rescued by reducing miR-3 levels, thereby directly implicating miRNA dysregulation in this aspect of the Dora deficiency phenotype (Kingston et al. 2022). In mice, loss of ZSWIM8 results in reduced body size, cardiopulmonary and neurodevelopmental defects, and perinatal lethality (Jones et al. 2023; Shi et al. 2023; Wang et al. 2023). Small RNA sequencing of E18.5 embryonic mouse *Zswim8*^−*/*−^ tissues revealed >50 upregulated miRNAs, suggesting widespread regulation of miRNAs by TDMD during mammalian development (Jones et al. 2023; Shi et al. 2023). Reduced dosage of miR-322-5p and miR-503-5p, two miRNAs that are cotranscribed as part of a miRNA cluster and share a 6 nt core seed sequence, rescued embryonic growth deficiency in *Zswim8*^−*/*−^ embryos, confirming that this phenotype is caused by aberrant overexpression of these miRNAs (Jones et al. 2023).

While there now exists an extensive catalog of miRNAs that are regulated by ZSWIM8 orthologs and are therefore inferred to be TDMD substrates, the presumptive trigger RNAs that are responsible for initiating degradation of these miRNAs have proven more difficult to identify. In *Drosophila*, bioinformatic analyses seeking conserved miRNA binding sites with extensive 3′ complementarity led to the identification of six TDMD trigger sites that function in S2 cells or fly embryos (Kingston et al. 2022). Recently, AGO cross-linking and sequencing of hybrids (AGO-CLASH) to identify AGO-associated miRNAs and their bound target RNAs (Helwak and Tollervey 2014) has emerged as a complementary tool to identify TDMD triggers. For example, AGO1-CLASH in *Drosophila* S2 cells led to the identification of the same TDMD triggers found by Kingston et al. (2022), supporting the use of this approach for identifying new triggers in additional settings (Sheng et al. 2023).

The vast majority of presumptive trigger RNAs in mammals remain to be defined. Despite the existence of >50 known miRNAs that are destabilized by ZSWIM8 in mouse tissues (Jones et al. 2023; Shi et al. 2023), as well as bioinformatic predictions of hundreds of potential mammalian trigger sites (Simeone et al. 2022), only four endogenous trigger sites have been validated thus far (Bitetti et al. 2018; Ghini et al. 2018; Kleaveland et al. 2018; Li et al. 2021). The most recently discovered mammalian TDMD trigger, *BCL2L11*, was discovered by analyzing AGO-CLASH data generated using mammalian cell lines and tissues (Li et al. 2021). Given recent advances in the AGO-CLASH methodology that enable the enhanced recovery of chimeras containing specific miRNAs of interest (Manakov et al. 2022), we reasoned that additional AGO-CLASH experiments focused on miRNAs that are strongly regulated by ZSWIM8 in mouse tissues might uncover additional TDMD triggers. Here we report the successful use of this approach to identify trigger sites that are required for TDMD of miR-322-5p and miR-503-5p in mouse embryonic fibroblasts (MEFs) (*Plagl1*:miR-322-5p, *Lrrc58*:miR-503-5p, and *Malat1*:miR-503-5p). In mice, deletion of the miR-322-5p and miR-503-5p trigger sites in *Plagl1* and *Lrrc58* impaired TDMD of these miRNAs in vivo and resulted in embryonic growth deficiency, partially phenocopying a key characteristic of *Zswim8*^−*/*−^ mice. This study therefore provides a valuable data set that will facilitate identification of additional mammalian TDMD triggers and establishes the existence of a *Plagl1*/*Lrrc58*-mediated TDMD pathway that plays a major role in regulating mammalian body size.

## Results

### AGO-CLASH identifies potential TDMD trigger RNAs

**Figure 1.**
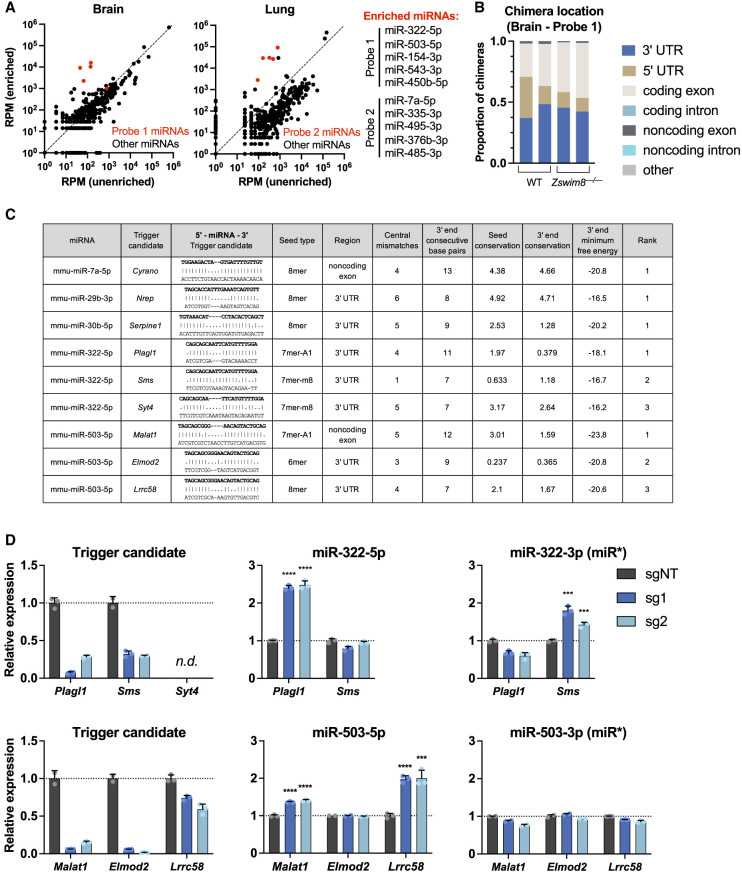
AGO-CLASH identifies potential TDMD trigger RNAs. (*A*) Plots showing reads per million (RPM) of the sum of all chimeras detected for each miRNA in enriched versus unenriched AGO-CLASH brain and lung samples. Enriched miRNAs are shown in red. (*B*) Proportion of chimeras mapped to each location in replicate WT and *Zswim8*^−*/*−^ brain AGO-CLASH samples enriched with probe 1. Only chimeras in which the predicted base-pairing to the miRNA seed sequence was a 6mer, 7mer-A1, 7mer-m8, or 8mer were included in the analysis. (*C*) Table of filtered and ranked candidate TDMD trigger sites identified by AGO-CLASH. Shown are the top-ranked triggers for miR-7a-5p, miR-29b-3p, and miR-30b-5p, and the top three candidate triggers for miR-322-5p and miR-503-5p. (*D*) qRT-PCR analysis of candidate trigger sites in MEFs. Immortalized MEFs expressing dCas9-KRAB were infected with lentivirus encoding a nontargeting guide (sgNT) or two independent guides targeting candidate trigger RNAs (sg1 and sg2). Shown is the candidate trigger expression normalized to *Actb* (*left*), mature miRNA abundance normalized to miR-16-5p (*middle*), and the passenger strand (miR*) levels normalized to miR-16-5p (*right*). Values were normalized to expression level in sgNT for each transcript. *n* = 3 technical replicates per sgRNA, with individual data points plotted (mean ± SD shown). *P*-values were calculated by one-tailed Student's *t*-test comparing sg1 or sg2 with sgNT. (***) *P* < 0.001, (****) *P* < 0.0001, (n.d.) not reliably detected.

To identify new TDMD triggers, we focused on a set of 10 miRNAs that are strongly regulated by ZSWIM8 in both mouse tissues and contact-inhibited MEFs (Supplemental Fig. S1A; Han et al. 2020; Jones et al. 2023; Shi et al. 2020, 2023). This set included miR-7a-5p as a positive control because its trigger RNA, *Cyrano* (also known as *Oip5os1*), has been identified (Kleaveland et al. 2018). We also included miR-322-5p and miR-503-5p because of their documented roles in regulating mammalian body size (Jones et al. 2023). A limitation of the traditional AGO-CLASH method is that it produces chimeric reads that are heavily biased toward highly abundant miRNAs and their targets. We therefore applied a recently developed AGO-CLASH protocol, also known as chimeric eCLIP, that incorporates an enrichment step using biotinylated probes to increase the representation of chimeras containing specific miRNAs of interest (Manakov et al. 2022). Two probes were designed to enrich for five miRNAs each (Fig. 1A; Supplemental Fig. S1A), and libraries were prepared using brain and lung tissue from wild-type and *Zswim8*^−*/*−^ mice (Jones et al. 2023). Chimeric reads represented a relatively small fraction of total reads (Supplemental Fig. 1B), as is typical for AGO-CLASH experiments. Nevertheless, a substantial increase in chimeric reads corresponding to the enriched miRNAs was observed in both the brain and lungs (Fig. 1A). Approximately 5%–40% of chimeric reads were comprised of a miRNA and a target RNA with a corresponding seed match (Supplemental Fig. 1B). A large majority of these sites mapped to 3′ UTRs and coding sequences (Fig. 1B; Supplemental Fig. 1C), consistent with the known locations of bona fide miRNA binding sites (Bartel 2018).

Chimeric reads in these data sets are expected to predominantly correspond to canonical miRNA target sites, with only a small fraction representing TDMD trigger sites. Therefore, as an initial approach to nominate sites that are most likely to function as TDMD triggers, we developed a set of highly stringent filtering criteria based on the features exemplified by the existing collection of validated *Drosophila*, mammalian, and viral trigger RNAs (Buhagiar and Kleaveland 2024). First, we mandated the presence of a seed match that was classified as a 6mer, 7mer-A1, 7mer-m8, or 8mer (Lewis et al. 2005; Grimson et al. 2007), excluding any potential triggers with mismatches within the seed binding sequence. Second, we selected only those candidate sites that fell within noncoding RNAs or within noncoding regions of mRNAs (5′ or 3′ UTRs) based on the observation that translating ribosomes diminish TDMD when the trigger site is located within an open reading frame (Li et al. 2025). Third, we excluded any potential trigger sites with large central bulges, defined as more than seven consecutive mismatches between the putative trigger and the 3′ sequence of the miRNA after the seed. Fourth, we required the presence of at least six consecutive base pairs between the trigger and the 3′ region of the miRNA (defined as the final 13 nt of the miRNA). Although mutagenesis experiments suggest that this latter feature is not absolutely required for TDMD (Han et al. 2020), all 13 validated TDMD triggers exhibit this attribute (Buhagiar and Kleaveland 2024). Fifth, we selected candidates that exhibit detectable evolutionary conservation of the seed binding region and the nucleotides predicted to base-pair with the 3′ region of the miRNA, as defined by a positive PhyloP (60 vertebrate) score (Pollard et al. 2010; Perez et al. 2025). Last, the minimum free binding energy of the predicted duplex between the potential trigger and 3′ region of the miRNA (3′ MFE) was calculated, and candidates were ranked based on this metric. Importantly, this pipeline ranked the known TDMD trigger sites in *Cyrano*, *Nrep*, and *Serpine1* as the top candidates for their respective miRNAs: miR-7a-5p, miR-29b-3p, and miR-30b-5p (Fig. 1C). Thus, although these criteria are not expected to capture all TDMD triggers due to their stringency, they provided a tractable set of promising candidates for initial validation.

### Validation of novel TDMD trigger RNAs

For eight of the enriched miRNAs in our AGO-CLASH experiments, we selected the top three most highly ranked candidate trigger sites for further validation (Fig. 1C; Supplemental Table S1). In one case, miR-450b-5p, only one candidate trigger (*Rdx*) remained after applying all criteria. Given the well-established role of *Cyrano* as a trigger for TDMD of miR-7a-5p (Kleaveland et al. 2018), additional candidates for this miRNA were not tested. The chimeras detected for candidate trigger sites spanned a wide range of abundances in our AGO-CLASH data sets (Supplemental Fig. 2A,B).

As an initial screen, CRISPR interference (CRISPRi) was used to knock down putative triggers in contact-inhibited immortalized MEFs (Gilbert et al. 2014). Previous work established that many ZSWIM8-regulated miRNAs are detectable in MEFs after contact inhibition, likely due at least in part to cessation of cell division, which reduces the dilution of stabilized miRNAs (Shi et al. 2020). Knockdown of *Cyrano* resulted in the expected increase in miR-7a-5p levels without affecting expression of the miR-7 passenger strand (miR-7a-3p), confirming the ability of this system to detect validated trigger activity (Supplemental Fig. 3A,B). We next knocked down each candidate trigger using two distinct single-guide RNAs (sgRNAs) and assessed the levels of the trigger, the target miRNA, and its respective passenger strand using qRT-PCR (Fig. 1D; Supplemental Fig. 3C). In cases where potential triggers were not reliably detected in MEFs (*Syt4*, *Lcp2*, *Ildr2*, *Frem1*, *Gm15477*, *Adam22*, and *Ccr2*), corresponding measurements of the miRNAs were not performed, with the reasoning that these transcripts could not be responsible for TDMD in this cell type.

Potential triggers for which knockdown with both guides resulted in a statistically significant increase in the respective miRNA without an increase in abundance of the passenger strand were selected for further validation. Importantly, this screening approach may yield false negatives, as residual transcripts remaining after CRISPRi-mediated knockdown may be sufficient to carry out TDMD in some cases. Additionally, if multiple redundant triggers exist for a single miRNA, knockdown of one may not be sufficient to result in detectable miRNA derepression. Nevertheless, even with these caveats, we were able to identify six potential trigger RNAs for four miRNAs of interest (Fig. 1D; Supplemental Fig. 3C). This set included the pairs *Plagl1*:miR-322-5p, *Malat1*:miR-503-5p, *Lrrc58*:miR-503-5p, *Lpar4*:miR-335-3p, *Dnal1*:miR-335-3p, and *Rdx*:miR-450b-5p. Knockdown of the top three candidate triggers for the other miRNAs tested (miR-154-3p, miR-495-3p, miR-485-3p, miR-543-3p, and miR-376b-3p) did not reproducibly increase the levels of the corresponding miRNAs (Supplemental Fig. 3C).

**Figure 2.**
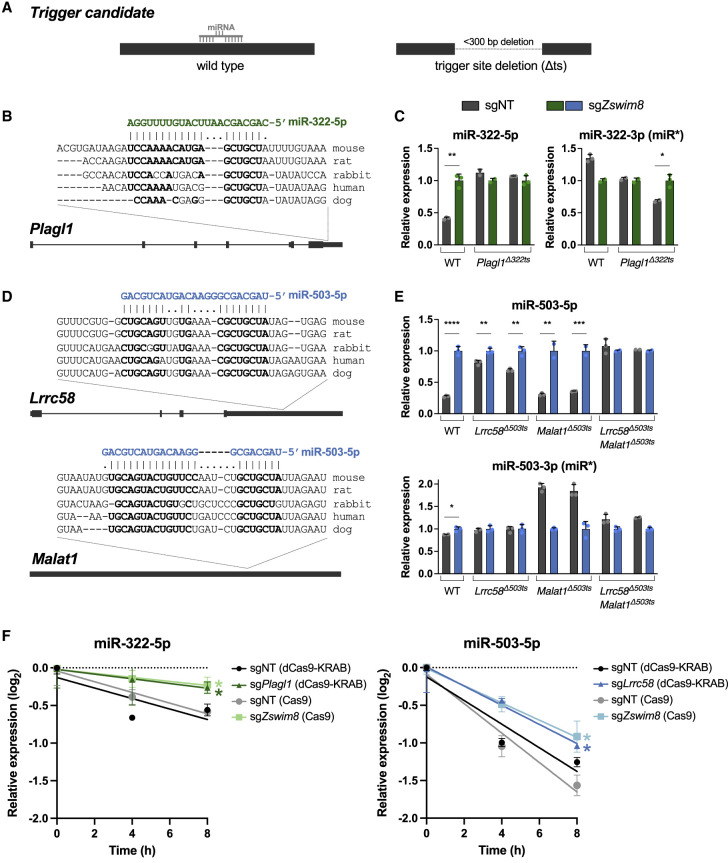
Validation of novel TDMD triggers. (*A*) Schematic of trigger site deletion (Δts) strategy to validate putative TDMD triggers. (*B*,*D*) Genomic organization of TDMD trigger transcripts with conservation and predicted miRNA base-pairing architecture of the trigger sites. Nucleotides predicted to base-pair with the miRNA are shown in bold. (*C*,*E*) qRT-PCR analysis of indicated miRNAs, relative to miR-16-5p, in WT and Δts MEFs. Parental MEFs or two independent Δts clones for each trigger site were infected with lentivirus expressing Cas9 and a nontargeting CRISPR guide (sgNT) or *Zswim8-*targeting guide (sg*Zswim8*). Values were normalized to expression level in sg*Zswim8* for each condition. *n* = 3 technical replicates per clone, with individual data points plotted (mean ± SD shown). *P*-values were calculated by one-tailed Student's *t*-test. (*) *P* < 0.05, (**) *P* < 0.01, (***) *P* < 0.001, (****) *P* < 0.0001. (*F*) qRT-PCR analysis of the indicated miRNAs, relative to U6, in NIH3T3 cells at time points after serum starvation and restimulation. NIH3T3 cells expressing dCas9-KRAB were infected with lentivirus encoding a nontargeting guide (sgNT) or guides targeting *Plagl1* or *Lrrc58*. Parental NIH3T3 cells were infected with lentivirus expressing Cas9 and a nontargeting guide (sgNT) or *Zswim8-*targeting guide (sg*Zswim8*). Values were normalized to expression at the 0 h time point for each condition. *n* = 3 technical replicates per condition, with mean ± SD shown. *P*-values were calculated by one-tailed Student's *t*-test comparing sg*Plagl1* (dCas9-KRAB) or sg*Lrrc58* (dCas9-KRAB) with sgNT (dCas9-KRAB) or comparing sg*Zswim8* (Cas9) with sgNT (Cas9). (*) *P* < 0.05.

To further validate potential triggers supported by CRISPRi experiments, CRISPR/Cas9-mediated genome editing was used to generate clonal MEF cell lines harboring deletions of each endogenous candidate trigger site (Fig. 2A; Supplemental Fig. S4A). These deletions were ∼100–300 bp in length and were located in 3′ UTRs in all cases except for the site in the noncoding RNA *Malat1*. To test whether loss of the putative trigger site abolished TDMD of the respective miRNA, knockout cell lines were infected with control or *Zswim8*-targeting lentiviral CRISPR vectors, and the resulting effects on miRNA expression were assessed after contact inhibition. As expected, loss of ZSWIM8 in wild-type MEFs led to an increase in the levels of all tested miRNAs, without a corresponding increase in the passenger strands (Fig. 2B–E; Supplemental Fig. S4B–E). In contrast, deletion of multiple individual candidate trigger sites impaired TDMD of their respective miRNAs. Specifically, loss of the miR-322-5p binding site in the 3′ UTR of *Plagl1* fully abrogated regulation of this miRNA by ZSWIM8, providing strong evidence that this transcript represents the sole trigger for miR-322-5p in MEFs (Fig. 2B,C). In the case of miR-503-5p, deletion of the putative trigger site in *Lrrc58* partially inhibited TDMD of this miRNA, while removal of the site in *Malat1* had no detectable effect (Fig. 2D,E). Combined deletion of these trigger sites, however, fully abolished TDMD of miR-503-5p, demonstrating that these transcripts act redundantly to induce decay of this miRNA in MEFs, with *Lrrc58* apparently acting as the dominant trigger. To our knowledge, this represents the first known example of a case in which two TDMD triggers can act on a single miRNA. Of note, human miR-503-5p differs from the mouse sequence by 1 nt (Supplemental Fig. S4F). Interestingly, there is a corresponding nucleotide change in the putative miR-503-5p binding site in the 3′ UTR of human *LRRC58*, suggesting selective pressure to maintain the base-pairing architecture of this site across species.

We also deleted putative trigger sites in *Lpar4* and *Dnal1* for miR-335-3p (alone and in combination) and in *Rdx* for miR-450b-5p, but these deletions did not impair TDMD of these miRNAs (Supplemental Fig. S4B–E). Therefore, these sites either do not function as TDMD triggers or act redundantly with other sites to induce decay of these miRNAs. Notably, the abundance of a chimera in the AGO-CLASH data was not a good predictor of its ultimate validation as a bona fide TDMD trigger site, as several highly represented chimeras failed to validate, while the trigger sites in *Cyrano*, *Plagl1*, *Malat1*, and *Lrrc58* exhibited a broad range of abundances across samples (Supplemental Fig. S2A,B). This pattern differed from the results of recently reported AGO-CLASH experiments in *Drosophila* S2 cells, where validated TDMD triggers often represented the most abundantly detected chimera (Hiers et al. 2025). Similarly, the expression of validated trigger transcripts in MEFs was not significantly higher than candidate triggers that failed to validate (Supplemental Fig. S2C).

ZSWIM8-dependent decay of miR-322-5p and miR-503-5p has been documented in NIH3T3 cells upon re-entry into the cell cycle after serum starvation-induced arrest (Rissland et al. 2011; Shi et al. 2020). To test whether the trigger sites in *Plagl1* and *Lrrc58* are responsible for this accelerated decay, we measured miR-322-5p and miR-503-5p levels after serum starvation and restimulation of NIH3T3 cells following CRISPRi-mediated knockdown of *Plagl1* or *Lrrc58*. Depletion of these transcripts stabilized both miRNAs to a magnitude equivalent to that observed upon knockout of *Zswim8*, demonstrating that these triggers are responsible for TDMD of these miRNAs following cell cycle re-entry (Fig. 2F). Altogether, these experiments identify *Plagl1*, *Lrrc58*, and *Malat1* as functional TDMD triggers in MEFs.

### Mice lacking the TDMD trigger sites in *Plagl1* and *Lrrc58* exhibit embryonic growth restriction

Germline deletion of *Zswim8* in mice results in perinatal lethality, heart and lung defects, and reduced body size at E18.5 (Jones et al. 2023; Shi et al. 2023). Deletion of miR-322 and miR-503 in *Zswim8*^−*/*−^ mice rescued embryonic growth (Jones et al. 2023), strongly suggesting that this aspect of the ZSWIM8 deficiency phenotype is attributable to aberrant upregulation of these miRNAs due to inactivation of TDMD. Interestingly, while the newly defined miR-503-5p triggers *Lrrc58* and *Malat1* have not previously been linked to regulation of embryonic growth in mammals, the miR-322-5p trigger *Plagl1* has been implicated as a regulator of body size in mice (Varrault et al. 2006). *Plagl1* (also known as *Zac1*) is a maternally imprinted gene that encodes a transcription factor that promotes growth by transactivating *Igf2* expression. A previously generated *Plagl1* knockout allele that eliminates expression of both the open reading frame and the 3′ UTR, where the miR-322-5p trigger site is located, results in embryonic growth restriction in mice, mirroring a key attribute of *Zswim8*^−*/*−^ mice (Varrault et al. 2006). These observations suggested that *Plagl1* may regulate body size through both the activity of the encoded protein and, potentially, the miR-322-5p TDMD trigger site in the mRNA.

**Figure 3.**
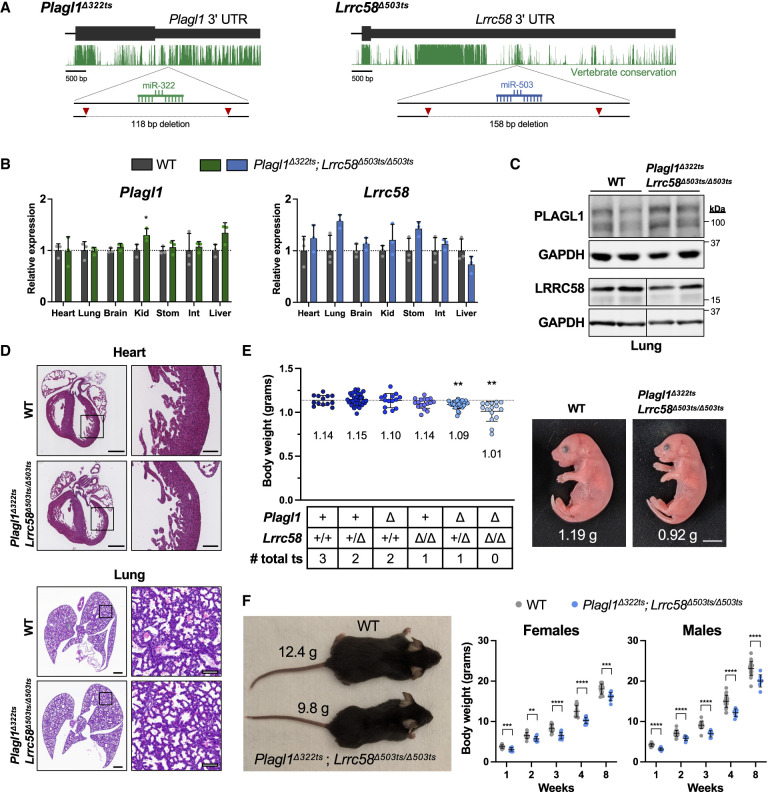
Embryonic growth restriction in *Plagl1*^*Δ322ts*^; *Lrrc58*^*Δ503ts/Δ503ts*^ mice. (*A*) Schematic of genome-editing strategy to generate *Plagl1*^*Δ322ts*^ and *Lrrc58*^*Δ503ts*^ mice. UCSC genome browser PhastCons 60 vertebrate conservation track shown (mm10). Red triangles depict approximate locations of sgRNAs for CRISPR-mediated editing. (*B*) qRT-PCR analysis of *Plagl1* and *Lrrc58* expression in E18.5 mouse tissues normalized to the geometric mean of two housekeeping genes (*Psmd4* and *Oaz1*). Expression was normalized to mean expression in WT in each tissue. *n* = 3 mice per genotype, with each mouse represented by an individual data point (mean ± SD shown). *P*-values were calculated by one-tailed Student's *t*-test comparing *Plagl1*^*Δ322ts*^; *Lrrc58*^*Δ503ts/Δ503ts*^ with WT for each tissue. (Kid) Kidney, (Stom) stomach, (Int) small intestine. (*C*) Western blot analysis of PLAGL1 and LRRC58 in E18.5 lung tissues of mice of the indicated genotypes. Irrelevant lanes were removed from blots where indicated with vertical lines. (*D*) Representative hematoxylin and eosin (H&E)-stained sections of E18.5 hearts and lungs from mice of the indicated genotypes. *n* = 4 mice analyzed per genotype. Scale bars: *left*, 500 µm; *right*, 100 µm. (*E*, *left*) Body weights of E18.5 embryos of the indicated genotypes, with each data point representing an individual mouse (mean ± SD shown). Mean weight is denoted in the graph *below* each cohort. The dotted line is the mean weight of the WT cohort. *n* = 14–32 mice per genotype. *P*-values were calculated by one-tailed Student's *t*-test comparing each genotype with WT. (*Right*) Image of littermate WT and *Plagl1*^*Δ322ts*^; *Lrrc58*^*Δ503ts/Δ503ts*^ E18.5 embryos, with weights indicated. Scale bar, 0.5 cm. (ts) Trigger site. (*F*, *left*) Image of littermate 4 week old male WT and *Plagl1*^*Δ322ts*^; *Lrrc58*^*Δ503ts/Δ503ts*^ mice, with body weights indicated. (*Right*) Graph of body weights of WT and *Plagl1*^*Δ322ts*^; *Lrrc58*^*Δ503ts/Δ503ts*^ mice at the indicated time points (mean ± SD shown). *n* = 8–22 mice for each genotype at each time point. *P*-values were calculated by one-tailed Student's *t*-test. (*) *P* < 0.05, (**) *P* < 0.01, (***) *P* < 0.001, (****) *P* < 0.0001.

To test this possibility, we generated mice with small deletions that remove the trigger sites from the 3′ UTRs of *Plagl1* (referred to as *Plagl1*^*Δ322ts*^) and *Lrrc58* (*Lrrc58*^*Δ503ts*^) (Fig. 3A). Given that the *Malat1* trigger site only had a small effect on miR-503 levels exclusively in MEFs lacking the *Lrrc58* trigger site, we elected to examine only *Lrrc58* as a potential trigger in vivo. Because *Plagl1* is a maternally imprinted gene with exclusive expression of the paternal allele (Spengler et al. 1997; Varrault et al. 2006), functionally heterozygous mice cannot be generated. We therefore denote the *Plagl1* genotype solely based on the identity of the inherited paternal allele (*Plagl1*^*+*^ or *Plagl1*^*Δ322ts*^). *Plagl1* and *Lrrc58* are widely expressed in mouse tissues, albeit at highly variable levels (Supplemental Fig. S5A), consistent with their potential to function as triggers for TDMD of miR-322-5p and miR-503-5p, which are broadly regulated by ZSWIM8 (Supplemental Fig. S1A; Jones et al. 2023; Shi et al. 2023). Notably, alternative splicing in the final exon of *Plagl1* produces transcript variants that contain or lack the miR-322-5p trigger site (Supplemental Fig. S5B).

In most tissues, the *Plagl1* trigger site deletion did not affect the steady-state abundance of the individual alternatively spliced *Plagl1* isoforms (Supplemental Fig. S5B) or the overall level of *Plagl1* transcripts (Fig. 3B), although a small increase (<1.5 fold) was observed in selected tissues. A modest but reproducible increase in PLAGL1 protein in the lung and heart, but not in MEFs, from *Plagl1*^*Δ322ts*^ mice was also detectable (Fig. 3C; Supplemental Fig. S5C). These findings suggested that the miR-322-5p binding site might confer canonical miRNA-mediated silencing in addition to functioning as a TDMD trigger site in some contexts. The *Lrrc58* trigger site deletion did not significantly affect expression of the mRNA or protein produced from the mutant locus in any tested tissue (Fig. 3B,C; Supplemental Fig. S5C).

Because miR-322-5p and miR-503-5p have the same core 6 nt seed sequence and therefore are expected to share highly overlapping targets, we examined the phenotypes of both single (*Plagl1*^*Δ322ts*^ or *Lrrc58*^*Δ503ts/Δ503ts*^) and double (*Plagl1*^*Δ322ts*^; *Lrrc58*^*Δ503ts/Δ503ts*^) trigger site knockout mice. Mice carrying all combinations of these alleles were viable and were born at the expected Mendelian ratios (Supplemental Fig. S5D). Accordingly, and in contrast to *Zswim8*^−*/*−^ mice, no overt abnormalities of the heart or lungs were apparent at E18.5 (Fig. 3D). However, the body size of E18.5 embryos was significantly attenuated in mice carrying trigger site deletions. Specifically, *Plagl1*^*Δ322ts*^; *Lrrc58*^*Δ503ts/Δ503ts*^ double-knockout mice and, to a lesser extent, *Plagl1*^*Δ322ts*^; *Lrrc58*^+*/Δ503ts*^ mice exhibited a statistically significant reduction in embryonic growth (Fig. 3E; Supplemental Fig. S5E). On average, mice lacking both trigger sites were ∼11% smaller than wild-type controls (Fig. 3E). In comparison, *Zswim8*^−*/*−^ mice were ∼22% smaller than control animals at this developmental time point (Jones et al. 2023; Shi et al. 2023), indicating that stabilization of miR-322-5p and miR-503-5p can account for about half of the growth defect characteristic of ZSWIM8-deficient mice. Interestingly, while the reduced body size of *Plagl1*^*Δ322ts*^; *Lrrc58*^*Δ503ts/Δ503ts*^ double-knockout mice was maintained into early adulthood (Fig. 3F), the relative postnatal growth rate of these mice was indistinguishable from wild-type mice (Supplemental Fig. S5F). This observation suggests that the growth restriction of *Plagl1*^*Δ322ts*^; *Lrrc58*^*Δ503ts/Δ503ts*^ mice occurs earlier in development, after which mutant animals grow at a normal rate. These results provide strong evidence that the TDMD trigger sites in the 3′ UTRs of *Plagl1* and *Lrrc58* cooperate to control embryonic growth in mice.

### The trigger sites in *Plagl1* and *Lrrc58* mediate TDMD of miR-322-5p and miR-503-5p in vivo

**Figure 4.**
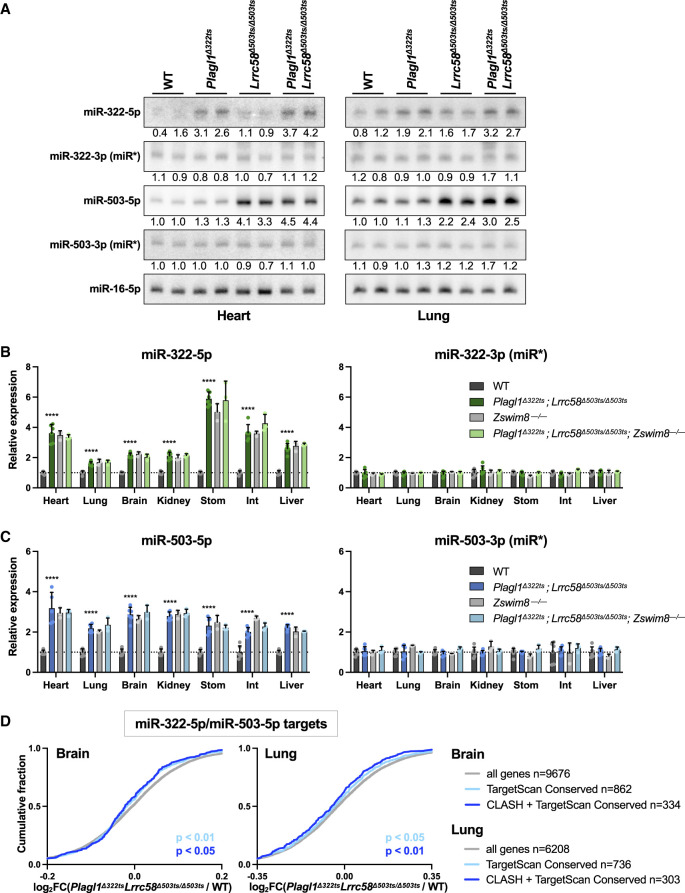
Loss of the *Plagl1* and *Lrrc58* trigger sites abrogates TDMD of miR-322-5p and miR-503-5p in vivo. (*A*) Northern blot analysis of miRNA expression in E18.5 hearts (*left*) and lungs (*right*) in mice of the indicated genotypes. Quantification relative to miR-16-5p, normalized to mean expression in WT, is shown *below* each lane. (*B*,*C*) qRT-PCR analysis of miRNAs (*left*) and passenger strands (*right*) normalized to miR-16-5p in mouse tissues of the indicated genotypes at E18.5. Expression in all genotypes was normalized to mean expression in WT in each tissue. *n* = 3–6 mice per genotype, with each mouse represented by an individual data point (mean ± SD shown). *P*-values were calculated by one-tailed Student's *t*-test comparing *Plagl1*^*Δ322ts*^; *Lrrc58*^*Δ503ts/Δ503ts*^ with WT for each tissue. (Stom) Stomach, (Int) small intestine. (****) *P* < 0.0001. (*D*) Cumulative distribution function (CDF) plot showing the fold change in expression of the following sets of mRNAs in the brain (*left*) and lungs (*right*) at E18.5, comparing *Plagl1*^*Δ322ts*^; *Lrrc58*^*Δ503ts/Δ503ts*^ with WT: (1) all genes with counts per million (CPM) >5 (gray), (2) the set of conserved targets of miR-322-5p or miR-503-5p as predicted by TargetScan (light blue) (McGeary et al. 2019), and (3) the set of conserved TargetScan-predicted targets that were also detected as chimeras with miR-322-5p or miR-503-5p in AGO-CLASH experiments (dark blue). *P*-values were calculated by one-sided Wilcoxon rank sum test.

We next examined levels of miR-322-5p and miR-503-5p and their corresponding passenger strands in tissues from *Plagl1* and *Lrrc58* trigger site knockout mice. Northern blotting of RNA from the heart and lungs confirmed that loss of the *Plagl1* trigger site specifically derepressed miR-322-5p but not its passenger strand (miR-322-3p), while deletion of the *Lrrc58* trigger site resulted in a specific accumulation of miR-503-5p but not its passenger (miR-503-3p) (Fig. 4A). Analysis of miRNA levels in livers from all combinations of *Plagl1*^*Δ322ts*^ and *Lrrc58*^*Δ503ts*^ genotypes further confirmed the specificity of *Plagl1* and *Lrrc58* trigger sites for their respective miRNAs and documented that the miR-322/503 passenger strands and pri-miRNA were insensitive to trigger site deletions in this tissue (Supplemental Fig. S6A). Similar results were observed in primary MEFs from *Plagl1*^*Δ322ts*^; *Lrrc58*^*Δ503ts/Δ503ts*^ double-knockout mice (Supplemental Fig. S6B). Expanding these analyses to a broad panel of tissues (heart, lung, brain, kidney, stomach, small intestine, and liver) demonstrated that, in all contexts, both miR-322-5p and miR-503-5p were upregulated in *Plagl1*^*Δ322ts*^; *Lrrc58*^*Δ503ts/Δ503ts*^ mice to a magnitude equivalent to that observed upon knockout of *Zswim8*, while passenger strand levels were unaffected in all tissues (Fig. 4B,C). Moreover, loss of *Zswim8* in trigger site knockout animals (*Plagl1*^*Δ322ts*^; *Lrrc58*^*Δ503ts/Δ503ts*^; *Zswim8*^−*/*−^) did not result in any further increase in miR-322-5p or miR-503-5p levels, providing strong evidence that *Plagl1* and *Lrrc58* are the sole triggers for these miRNAs in these tissues. While these data argue against a role for *Malat1* as a TDMD trigger for miR-503-5p in these contexts, it remains possible that *Malat1* performs this function in other cell types or time points in vivo.

To determine whether stabilization of miR-322-5p and miR-503-5p in trigger site knockout mice enhances repression of targets of these miRNAs, RNA sequencing was performed on tissues from E18.5 wild-type and *Plagl1*^*Δ322ts*^; *Lrrc58*^*Δ503ts/Δ503ts*^ mice. Consistent with the magnitude of target repression reported previously in *Zswim8*^−*/*−^ tissues (Shi et al. 2023), we observed a modest but significant enhancement of repression of miR-322-5p and miR-503-5p targets in the brains and lungs from *Plagl1*^*Δ322ts*^; *Lrrc58*^*Δ503ts/Δ503ts*^ animals (Fig. 4D). These data therefore establish that *Plagl1* and *Lrrc58* are functional triggers for TDMD of miR-322-5p and miR-503-5p in mouse tissues and likely represent the sole triggers for these miRNAs in the tissues examined at this developmental time point (E18.5).

### Growth restriction in *Plagl1* and *Lrrc58* trigger site knockout mice is miR-322/503-dependent

**Figure 5.**
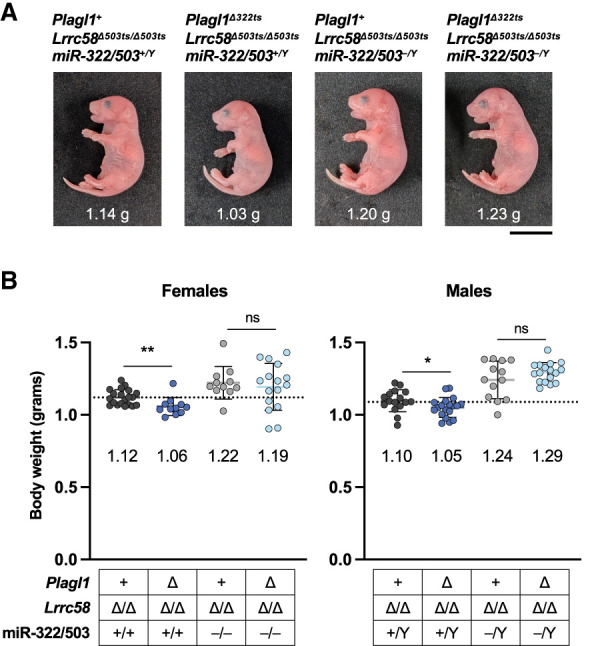
miR-322/503-dependent growth restriction in *Plagl1*^*Δ322ts*^; *Lrrc58*^*Δ503ts/Δ503ts*^ mice. (*A*) Images of E18.5 male mice of the indicated genotypes, with body weights shown. Scale bar, 1 cm. (*B*) Graphs of body weights of female and male E18.5 mice of the indicated genotypes, with individual weights plotted (mean ± SD shown). Mean weight is denoted *below* each cohort. The dotted line is the mean weight of the *Plagl^+^; Lrrc58*^*Δ503ts/Δ503ts*^ cohort. *n* = 11–20 mice per genotype. *P*-values were calculated by one-tailed Student's *t*-test. (*) *P* < 0.05, (**) *P* < 0.01, (ns) not significant.

To confirm that trigger site deletions in *Plagl1* and *Lrrc58* restrict embryonic growth in a manner dependent on miR-322-5p and miR-503-5p, we crossed *Plagl1*^*Δ322ts*^; *Lrrc58*^*Δ503ts/Δ503ts*^ mice to our previously reported miR-322/503^−/−^ mouse line (Jones et al. 2023). Because of the large number of alleles involved, we specifically compared *Plagl1*^+^*; Lrrc58*^*Δ503ts/Δ503ts*^ mice (whose body size is equivalent to wild type) (see Fig. 3E) with *Plagl1*^*Δ322ts*^; *Lrrc58*^*Δ503ts/Δ503ts*^ mice with or without an intact miR-322/503 locus present. In keeping with our findings described above, complete loss of the trigger sites resulted in reduced body size of male and female E18.5 embryos in a miR-322/503 wild-type background (Fig. 5A,B). As expected, deletion of miR-322/503 resulted in larger embryos, as we reported previously (Jones et al. 2023). Importantly, however, in miR-322/503-deficient mice, complete trigger site deletion did not reduce embryonic growth. Thus, embryonic growth restriction in *Plagl1*^*Δ322ts*^; *Lrrc58*^*Δ503ts/Δ503ts*^ mice requires miR-322-5p and miR-503-5p, providing strong evidence that this phenotype is attributable to loss of TDMD of these miRNAs in these animals.

## Discussion

ZSWIM8 broadly sculpts miRNA expression in metazoans and, accordingly, is essential for normal development in diverse species. Nevertheless, identification of the presumptive trigger RNAs that induce decay of many ZSWIM8-regulated miRNAs has proven to be a challenging problem. For example, miR-322-5p and miR-503-5p were among the earliest miRNAs found to exhibit accelerated decay kinetics (Rissland et al. 2011). While ZSWIM8 was later shown to be responsible for the lability of these miRNAs (Shi et al. 2020), the presumptive trigger RNAs that initiate their degradation have remained unknown. Here, using an enhanced AGO-CLASH method (Manakov et al. 2022) focused on the miRNAs that are most strongly regulated by ZSWIM8 in mouse tissues, we identified *Plagl1* and *Lrrc58* as the long-sought TDMD triggers for miR-322-5p and miR-503-5p. Moreover, we demonstrated that loss of these trigger sites in mice results in miR-322/503-dependent embryonic growth restriction, partially recapitulating a central feature of the ZSWIM8 deficiency phenotype. Together, these findings demonstrate that control of miRNA expression by TDMD is essential for normal development in mammals and reveal a noncoding function for the *Plagl1* and *Lrrc58* transcripts as regulators of mammalian body size.

As an initial approach to nominate candidate TDMD trigger sites among the many miRNA-binding sites represented in our AGO-CLASH data set, we applied a highly stringent filtering strategy that required a perfect seed match and extensive base-pairing between the miRNA 3′ end and the trigger. These criteria were modeled based on features displayed by most previously validated TDMD triggers (Buhagiar and Kleaveland 2024) and enabled identification of the miR-322-5p and miR-503-5p triggers. Nevertheless, this strategy was not sufficient to distinguish bona fide triggers from the many possible candidates for other ZSWIM8-regulated miRNAs that were enriched in our AGO-CLASH experiments. There are multiple possible reasons for this limitation that could be addressed in future studies. First, AGO-CLASH was performed using lung and brain tissues, while validation studies were performed in MEFs. It is possible that distinct triggers mediate TDMD in these different contexts. Second, it is likely that the stringent criteria that we applied excluded functional TDMD triggers. Our understanding of the base-pairing architectures that induce TDMD remains incomplete and may vary between different miRNAs. For example, mutagenesis of HSUR1, the herpesvirus-encoded trigger for TDMD of miR-27a, demonstrated that extensive complementarity with the miRNA 3′ end is required for activity of this miRNA:trigger pair (Cazalla et al. 2010; Sheu-Gruttadauria et al. 2019). However, there is at least one example of a miRNA family regulated by the ZSWIM8 homolog EBAX-1 in *C. elegans*—miR-35, whose degradation is specified only by the seed sequence (Donnelly et al. 2022)—and a recently discovered TDMD trigger in *Drosophila* requires only minimal 3′ pairing (Hiers et al. 2025). These findings demonstrate that some TDMD interactions do not require extensive 3′ base-pairing of the miRNA. Such triggers, should they exist in mammals, might be represented within our AGO-CLASH data set but would not be selected as candidate triggers using the bioinformatics approach applied here. Future experiments designed to systematically evaluate the base-pairing requirements for TDMD of individual miRNAs would aid future efforts to rationally nominate candidate trigger sites that engage in empirically determined base-pairing architectures with their cognate miRNAs.

Redundancy could also potentially confound the identification of new TDMD trigger sites. Indeed, in this study, we identified a case of two triggers acting on the same miRNA, which is, to our knowledge, the first documented example of this principle. While *Lrrc58* is the dominant trigger for TDMD of miR-503-5p, we observed that, at least in MEFs, there is a measurable contribution of a site in the noncoding RNA *Malat1* to TDMD of this miRNA. *Malat1* is a predominantly nuclear transcript, although recent studies have shown it can localize to the cytoplasm in select cell types (Xiao et al. 2024). Regardless, given the extremely high abundance of *Malat1*, localization of even a small fraction of the transcript to the cytoplasm might be sufficient for a productive TDMD interaction in this compartment. It is possible that in rapidly cycling cells such as MEFs, clearance of *Malat1* from the cytoplasm after mitosis may be incomplete. This might explain why *Malat1* measurably contributes to TDMD of miR-503-5p in MEFs but not in tissues with large populations of slowly dividing or terminally differentiated cells. The trigger site that we identified in *Malat1* has previously been shown to bind miR-15-5p and miR-16-5p, which share a core seed sequence with miR-503-5p, in mouse CD8^+^ T cells (Wheeler et al. 2023). Notably, the 3′ sequences of miR-15-5p/16-5p are distinct from miR-503-5p, likely explaining why *Malat1* does not induce TDMD of these miRNAs. Nevertheless, these data provide independent confirmation that this site is accessible to miRNA-mediated AGO binding. This example illustrates the potential for multiple triggers to regulate an individual miRNA, which might necessitate dual knockdown or knockout approaches to uncover the TDMD activity of some yet to be validated triggers.

Given our finding that loss of the trigger sites in both *Plagl1* and *Lrrc58* is required to manifest a significant defect in embryonic growth, we can conclude that miR-322-5p and miR-503-5p both function as negative regulators of body size and together contribute to the small body size of ZSWIM8-deficient mice. This is not unexpected in light of the shared core seed sequence and coregulation of these clustered miRNAs. Together, stabilization of these miRNAs can account for approximately half of the growth defect characteristic of *Zswim8*^−*/*−^ mice (growth defect of ∼11% in *Plagl1*^*Δ322ts*^; *Lrrc58*^*Δ503ts/Δ503ts*^ mice vs. ∼22% in *Zswim8*^−*/*−^ mice) (Jones et al. 2023; Shi et al. 2023). The stronger growth restriction observed in *Zswim8*^−*/*−^ mice could be due to stabilization of other TDMD-regulated miRNAs with growth-suppressing activity in these animals or may be a consequence of the other developmental abnormalities caused by ZSWIM8 loss. For example, in humans, congenital heart defects are associated with low birth weight (Aliasi et al. 2023). Additionally, the small increase in PLAGL1 protein levels observed in some tissues of *Plagl1*^*Δ322ts*^ mice might dampen the amplitude of growth restriction in trigger site knockout animals.

Why does accumulation of miR-322-5p and miR-503-5p lead to embryonic growth restriction? These mammalian-specific miRNAs have been reported to regulate diverse pathways, including insulin-like growth factor (IGF) signaling and the cell cycle, both of which play important roles in regulating mammalian body size (Conlon and Raff 1999; Linsley et al. 2007; Rissland et al. 2011; Yang and Xu 2011; Llobet-Navas et al. 2014). For example, *Igf1r*, whose loss results in severe growth restriction in mice (Liu et al. 1993), has been shown to be a target of miR-322-5p and miR-503-5p in mouse mammary epithelial cells (Llobet-Navas et al. 2014). Moreover, as members of the miR-16 family, miR-322-5p and miR-503-5p have the potential to target multiple proteins required for cell cycle progression (Linsley et al. 2007; Bandi et al. 2009; Jiang et al. 2009). Of particular relevance, loss of *Ccnd1*, which encodes cyclin D1 and is a validated target of miR-503-5p and other miR-16 family members, results in small body size in mice (Fantl et al. 1995; Sicinski et al. 1995). Thus, silencing of *Igfr1* and *Ccnd1* offers possible mechanisms linking miR-322-5p/503-5p upregulation to growth restriction. Importantly, despite their smaller body size at birth, the relative postnatal growth rate of *Plagl1*^*Δ322ts*^; *Lrrc58*^*Δ503ts/Δ503ts*^ mice is indistinguishable from that of wild-type animals. This finding indicates that overexpression of miR-322-5p/503-5p in trigger site knockout mice impacts embryonic growth at an earlier developmental stage, after which mutant animals grow normally. Consistent with this observation, *Plagl1* expression drops dramatically after birth, suggesting that TDMD of miR-322-5p might be less robust in postnatal tissues (Lui et al. 2008). Further study of trigger site mutant animals throughout embryogenesis is warranted in order to pinpoint the critical developmental window during which accelerated degradation of miR-322-5p/503-5p impacts growth and to identify the specific targets that mediate this effect.

Previous work revealed an example in which the presence of a TDMD trigger site in an mRNA enabled the congruent regulation of a single biological process through dual coding and noncoding functions of the transcript. Specifically, *BCL2L11* promotes apoptosis by encoding the proapoptotic protein BIM while also functioning as a TDMD trigger that removes the apoptosis-suppressing miRNAs miR-221/222 (Zhang et al. 2010; Sionov et al. 2015; Li et al. 2021). Our discovery that the *Plagl1* transcript contains a trigger site for TDMD of miR-322-5p provides another example of this concept. A previously generated knockout allele of *Plagl1*, which eliminates expression of both the protein-coding sequence and the 3′ UTR where the miR-322-5p trigger site is located, results in fetal growth restriction, altered bone formation, and incompletely penetrant lethality shortly after birth (Varrault et al. 2006). Although the molecular mechanisms underlying these phenotypes are not fully understood, PLAGL1 protein was shown to directly transactivate *Igf2* expression in mice and humans, the loss of which likely contributed to growth restriction in knockout animals (Varrault et al. 2006; Iglesias-Platas et al. 2014). Our data reveal a dual role for *Plagl1* in regulating body size via its canonical protein-coding function as well as through the ability of the transcript to directly eliminate expression of the growth-suppressing miRNA miR-322-5p. These dual functions may have contributed to the severity of the *Plagl1* knockout mouse phenotype. Interestingly, alternative splicing in the *Plagl1* 3′ UTR produces two isoforms, one of which lacks the miR-322-5p TDMD trigger site. This configuration provides a mechanism to decouple production of the PLAGL1 protein from expression of the TDMD trigger. Although we detected broad expression of both *Plagl1* isoforms in bulk mouse tissues at E18.5, it is possible that production of the protein along with high levels of miR-322-5p is advantageous in some settings, which could be achieved by selective expression of the TDMD-deficient *Plagl1* isoform. These observations set the stage for further exploration of the regulation and role of *Plagl1*/*Lrrc58*-mediated degradation of miR-322-5p/miR-503-5p in mammalian body size control.

## Materials and methods

### AGO-CLASH

#### Sample preparation

Mouse brains and lungs were harvested at E18.5, snap-frozen in liquid nitrogen, and stored at −80°C. Each sample used for AGO-CLASH consisted of lungs from two mice or a single mouse brain. For UV-cross-linking, frozen tissues were ground using a cold mortar and pestle equilibrated in liquid nitrogen, resuspended in 6 mL of 1× phosphate-buffered saline (PBS), and UV-cross-linked in a 10 cm tissue culture dish at 243 nm and 400 mJ/cm^2^. Cross-linked tissues were harvested by centrifugation at 400*g* for 2 min, snap-frozen in liquid nitrogen, and stored at −80°C until further use.

#### AGO immunoprecipitation, chimeric ligation, and sequencing library preparation

For AGO2 immunoprecipitation, 5 µg of AGO2 antibody (eIF2C2 4F9; Santa Cruz Biotechnology) was immobilized on 200 µL of sheep antimouse Dynabeads (Invitrogen). Cross-linked lung samples were lysed in 500 µL of iCLIP lysis buffer (50 mM Tris-HCl at pH 7.4, 100 mM NaCl, 1% NP-40, 0.1% SDS, 0.5% sodium deoxycholate, 5.5 µL of 200× protease inhibitor cocktail III [EMD Millipore], 11 µL of murine RNase inhibitor [New England Biolabs]), and cross-linked brain samples were lysed in 1 mL of iCLIP lysis buffer. Samples were incubated for 5 min on ice, followed by sonication using a Bioruptor (Diagenode) on “low” setting for 5 min at 4°C (30 sec on/30 sec off). After lysis, 5 or 10 µL of Turbo DNase (Thermo Fisher Scientific) was added to lung or brain samples, respectively, followed by the addition of 10 or 20 µL of 1:300 diluted RNase I (Thermo Fisher Scientific) in PBS, respectively. Lysates were then incubated for 5 min at 37°C with agitation at 1200 rpm using an Eppendorf ThermoMixer C. Lysates were cleared by centrifugation at 13,000*g* for 3 min at 4°C and then added to anti-AGO2 immobilized beads. After overnight incubation at 4°C, bead washes, T4 PNK Minus reaction, RNA chimeric ligation, FastAP treatment, PNK treatment, and ligation of 3′-RNA linker (roJH031 in this study) were conducted as described previously (Manakov et al. 2022). Following proteinase K (New England Biolabs) treatment, RNA isolation was performed using Zymo RNA Clean & Concentrator-5 columns. To enrich specific microRNAs, probe capture was conducted in brain tissues using oJH1023 (for mmu-miR-322-5p, 503-5p, 154-3p, 543-3p, and 450b-5p) and in brain and lung tissues using oJH1024 (for mmu-miR-335-3p, 495-3p, 376b-3p, 7a-5p, and 485-3p). Isolated chimeric RNAs from unenriched or enriched samples were subjected to cDNA synthesis using oJH1016/1017 followed by 5′ linker (oJH1018) ligation overnight and then cleaned up using Dynabeads MyOne Silane (Thermo Fisher Scientific). Sequencing libraries were prepared using Q5 PCR master mix (New England Biolabs) with Illumina NextSeq primers and 16–17 PCR cycles. PCR products were separated on a 2% agarose gel, and fragments between 190 and 350 bp in size were extracted from the gel and sequenced on an Illumina NextSeq 2K. At least 50 million reads were obtained for each sample. All oligonucleotide sequences are listed in Supplemental Table S2.

#### Sequencing data analysis

UMI barcodes were extracted from the reads using UMI-tools (v1.1.2) (Smith et al. 2017). Adapter sequences were trimmed with Cutadapt (v3.1) (Martin 2011). Reads corresponding to snoRNAs were removed by aligning trimmed reads to mouse snoRNAs using snoRNA Atlas (Jorjani et al. 2016) and Bowtie (v1.3.1) (Langmead et al. 2009). Remaining reads were then mapped to mature mouse miRNA sequences from miRBase v22 (Kozomara et al. 2019) using Bowtie. Reads were filtered based on strand orientation and mismatch rate using chim-eCLIP (https://github.com/YeoLab/chim-eCLIP), which was also used to identify candidate chimeric reads. Chimeric reads that aligned to repetitive elements from the mouse genome (Repbase v27.07) using STAR (v2.7.1a) (Dobin et al. 2013) were excluded from downstream analyses. The remaining reads were aligned to the mouse reference genome (GRCm38) using Bowtie, and duplicate reads were removed using UMI-tools based on UMI barcodes. Enriched peak clusters were called using CLIPper (https://github.com/YeoLab/clipper), with peaks extended by 10 nt at the 5′ end. Annotations were based on GENCODE M23. Conservation scores were calculated using phyloP-60way (Pollard et al. 2010), and the minimum free energy (MFE) of base-pairing between miRNAs and target sequences was calculated using RNAhybrid (Rehmsmeier et al. 2004).

### Cell culture

HEK293T and NIH3T3 cells were obtained from ATCC, and immortalized MEFs were generated previously by transfecting primary MEFs with a plasmid expressing SV40 large T antigen (Kopp et al. 2019). Cells were cultured in DMEM (high glucose, pyruvate) supplemented with 10% FBS (Sigma) and either 1× penicillin–streptomycin antibiotic (Thermo Fisher Scientific) or 1× antibiotic–antimycotic (Invitrogen). Cell lines were confirmed to be free of mycoplasma contamination.

### Primary MEF generation

Primary MEFs were isolated from E14.5 mouse embryos as described previously (Tan and Lei 2019). Briefly, E14.5 embryos were dissected, and the head, liver, and heart were removed. The remaining tissue was finely minced in cold 0.25% trypsin (Gibco), pipetted up and down 10 times with a 5 mL serological pipette, and then incubated for 10 min at 37°C. Cells were then strained through a 40 µm cell strainer, spun at 500*g* for 5 min at 4°C, resuspended in 5 mL of fresh DMEM with 10% (v/v) fetal bovine serum (Sigma) and 1× antibiotic–antimycotic (Invitrogen), and plated in a 60 mm tissue culture dish. Primary MEFs were grown for less than five passages.

### Lentiviral CRISPR and CRISPRi

Immortalized MEFs or NIH3T3 cells stably expressing dCas9-KRAB were generated by infecting cells with lentiviral construct pHR-SFFV-dCas9-BFP-KRAB (Addgene 46911) (Gilbert et al. 2014). For MEFs, single cells positive for BFP were sorted into 96 well plates using a Melody cell sorter (BD Biosciences), and clonal cell lines were established. For NIH3T3 cells, the top 10% brightest BFP-expressing cells were sorted. sgRNAs targeting candidate trigger RNAs, as well as nontarget (NT) sgRNAs (sequences listed in Supplemental Table S2), were cloned into pUC6-sgRNA EF1Alpha-puro-T2A-BFP (Addgene 60955) (Gilbert et al. 2014). Guide sequences were taken from the mCRISPRiv2 library (Horlbeck et al. 2016) or designed using CRISPick (Doench et al. 2016; Sanson et al. 2018). For pooled *Zswim8* knockout experiments, previously reported lentiCRISPRv2-hygro vectors expressing nontargeting or *Zswim8*-targeting guides were used (Han et al. 2020), and cells were contact-inhibited prior to harvesting.

To generate lentivirus, 0.5 × 10^6^ to 1 × 10^6^ HEK293T cells were seeded per well in a 6 well plate. Twenty-four hours after seeding, cells were transfected with 500 ng of lentiviral vector, 300 ng of psPAX2 (Addgene 12260), and 200 ng of pMD2.G (Addgene 12259) using 3 µL of FuGENE HD (Promega) transfection reagent. At 24 h after transfection, the medium was changed, and 48 h after transfection, viral supernatant was collected and passed through a 0.45 µm filter. Recipient cells were transduced with viral supernatant in the presence of 8 µg/mL polybrene (Millipore). Beginning 48 h after transduction, cells were selected in medium containing 2 µg/mL puromycin (Invivogen) or 200–400 µg/mL hygromycin B (Invitrogen) for 5 days. MEFs were contact-inhibited for 1–3 days prior to harvesting.

### Generation of trigger site knockout cell lines

MEF trigger site knockout clones were generated in one of two ways: (1) Guide sequences were cloned into px458 (Addgene 48138) (Ran et al. 2013) expressing GFP and Cas9, and immortalized MEFs were transfected with plasmids using FuGENE HD (Promega). Twenty-four hours after transfection, GFP-positive cells were sorted into 96 well plates using a Melody cell sorter (BD Biosciences), and clonal cell lines were established. (2) sgRNAs were synthesized (Integrated DNA Technologies), resuspended at a final concentration of 25 µM, and incubated with 25 µM Alt-R S.p. Cas9 nuclease V3 (Integrated DNA Technologies) in PBS for 15 min at room temperature. Electroporation enhancer (IDT) was added to a final concentration of 12 µM along with 0.6 µg of pMax GFP. The mixture was electroporated into immortalized MEFs using the Amaxa P3 primary cell kit (Lonza) with program EH-100 on a 4D Nucleofector X unit (Lonza). Seventy-two hours to 96 h after nucleofection, GFP-positive cells were sorted into 96 well plates, and clonal cell lines were established. Cell lines were screened for deletions by PCR and amplicon sequencing. Amplicon sequencing was analyzed using CRISPResso2 (Clement et al. 2019). sgRNA sequences are listed in Supplemental Table S2.

### RNA isolation and qRT-PCR

Total RNA was isolated using the miRNeasy mini kit (Qiagen) with DNase I digestion according to the manufacturer's instructions. For lysing cells, QIAzol (Qiagen) was added directly to tissue culture plates after rinsing with PBS. For E18.5 tissues, organs were homogenized in QIAzol (Qiagen) using a Precellys Evolution homogenizer (Bertin Technologies). cDNA was synthesized according to the manufacturer's instructions using Mir-X miRNA first strand synthesis kit (Takara) for miRNAs and U6 or PrimeScript RT (Takara) for all other transcripts. qRT-PCR was performed with SYBR Green Master mix (Applied Biosystems) on a QuantStudio 3 (Applied Biosystems). miR-16 or U6 snRNA was used to normalize qRT-PCR measurements of miRNA expression. *Actb* was used to normalize mRNA measurements in cell culture experiments, while the geometric mean of *Psmd4* and *Oaz1* was used to normalize mRNA expression in tissues (Rehfeld et al. 2018). Primer sequences are listed in Supplemental Table S2.

### Analysis of miRNA decay in serum-stimulated NIH3T3 cells

CRISPRi was performed in NIH3T3 cells stably expressing dCas9-KRAB by infecting with pUC6-sgRNA EF1Alpha-puro-T2A-BFP (Addgene 60955) (Gilbert et al. 2014) expressing sgNT, sg*Plagl1* (sg1), or sg*Lrrc58* (sg1). Parental NIH3T3 cells were infected with lentiCRISPR_v2-hygro vectors expressing sgNT or sg*Zswim8* (Han et al. 2020). Lentivirally infected cells were washed three times with 1× PBS and grown in DMEM containing 0.5% donor bovine serum (Gibco) for 48 h. After restimulation with DMEM containing 20% donor bovine serum, cells were harvested 0, 4, or 8 h later for analysis of RNA levels by qRT-PCR. sgRNA and primer sequences are listed in Supplemental Table S2.

### Generation and husbandry of genetically engineered mice

All mouse experiments were approved by the University of Texas Southwestern Medical Center Animal Care and Use Committee and performed in accordance with National Institutes of Health guidelines (animal protocol 2017-102001). Mice were group-housed under conventional conditions in a 12 h day/night cycle with normal chow diet (Harlan Teklad TD2916) and water provided ad libitum. *Zswim8*^−*/*−^ (Jax strain 039272) and miR-332/503^−*/*−^ mice (Jax strain 039271) were described previously (Jones et al. 2023).

*Plagl1*^*Δ322ts*^; *Lrrc58*^*Δ503ts/Δ503ts*^ mice were generated in the University of Texas Southwestern Transgenic Core by microinjection of Cas9 in complex with sgRNAs (Integrated DNA Technologies) into the pronucleus of fertilized C57BL/6J eggs. Sequences of sgRNAs are listed in Supplemental Table S2. Founders carrying the desired alleles were maintained on a pure C57BL/6J background and backcrossed continuously. For timed matings, the morning of detection of vaginal plug was defined as E0.5.

### Western blotting

Cells were lysed in RIPA buffer (150 mM NaCl, 1% NP-40, 0.5% sodium deoxycholate, 0.1% SDS, 25 mM Tris-HCl at pH 8.0), with 2× EDTA-free protease inhibitor cocktail (Roche) and 1 mM phenylmethylsulfonyl fluoride (Sigma). Mouse tissues were lysed in the same buffer using a Bioruptor Plus (Diagenode). Lysates were cleared by centrifugation. Proteins were separated by SDS-PAGE and transferred to 0.2 µm nitrocellulose membranes (Cytiva). Blots were blocked in TBS with 0.1% Tween-20 (TBST) containing 5% nonfat milk and incubated with primary antibodies in TBST containing 3% BSA overnight at 4°C. After washing in TBST, the blots were incubated with secondary antibodies in TBST with 3% BSA for 1 h at room temperature followed by further washes. Images were acquired on an Odyssey fluorescent imaging system (LI-COR Biosystems). Primary antibodies used were as follows: anti-HA (1:1000; Cell Signaling Technologies C29F4), anti-PLAGL1 (1:500; Santa Cruz Biotechnology 166944), anti-LRRC58 (1:100; Thermo Fisher PA5-63453), and anti-GAPDH (1:2000; Santa Cruz Biotechnology 32233). Secondary antibodies used were antimouse and antirabbit secondary IR dye 800CW (LI-COR Biosystems).

### Histology

Tissues were fixed in 10% formalin, embedded in paraffin, and sectioned to show maximum surface area of the lung or four-chambered view of the heart. Hematoxylin and eosin (H&E) staining was performed on paraffin sections. Slides were scanned using a Leica Aperio slide scanner, and images were generated in Aperio ImageScope (Leica Biosystems).

### Northern blotting

RNA isolation was performed as described above. For heart samples, two to three hearts of the same genotype were pooled for each lane. Twenty micrograms of RNA was separated on 15% TBE-urea polyacrylamide gels. RNAs were transferred to BrightStar-Plus nylon membranes (Invitrogen) followed by UV-cross-linking at 120 mJ/cm^2^. Membranes were prehybridized with ULTRAhyb-Oligo hybridization buffer (Invitrogen) followed by hybridization with ^32^P end-labeled oligonucleotide probes. A DNA oligonucleotide probe was used for miR-16-5p, while miRCURY LNA miRNA detection probes (Qiagen) were used for miR-322-5p, miR-322-3p, miR-503-5p, and miR-503-3p. Probe sequences are listed in Supplemental Table S2. Densitometry was performed using Quantity One 1-D analysis software (Bio-Rad), and miR-16-5p was used as a normalization control.

### RNA sequencing

RNA-seq libraries were constructed using the QuantSeq 3′ mRNA-seq V2 library preparation kit FWD with UDI 12 nt sets A1 and B1 (Lexogen 192.24) using 500 ng of input RNA. Libraries were sequenced on a NextSeq 2K (Illumina) with 65 bp single-end reads. Reads were mapped to the reference mouse genome (GRCm39) using STAR (v2.7.11b) (Dobin et al. 2013). Gene-level read counts were obtained using featureCounts (v1.6.0) (Liao et al. 2014) with Gencode M31 annotation (Frankish et al. 2023). Differential expression analysis was performed using the edgeR package (v4.2.1) (Chen et al. 2025).

### Conservation alignments

For base-level conservation analyses (Fig. 2; Supplemental Fig. S4), sequences were obtained from the following genome assemblies: *Mus musculus* GRCm38/mm10, *Rattus norvegicus* RGSC 6.0/rn6, *Oryctolagus cuniculus* Broad/oryCun2, *Homo sapiens* GRCh38/hg38, and *Canis lupus familiaris* UU_Cfam_GSD_1.0/canFam4. Conservation tracks were obtained from the UCSC genome browser (PhastCons 60 vertebrate conservation track) (Yang 1995; Felsenstein and Churchill 1996; Siepel et al. 2005; Pollard et al. 2010).

### Statistics

To determine statistical significance, one-tailed Student's *t*-test and χ^2^ test were calculated using GraphPad Prism (version 10.5.0), and Wilcoxon rank sum test with continuity correction was calculated using R. Values are reported as mean ± SD in all figures.

### Data access

All sequencing data generated in this study have been deposited in GEO (AGO-CLASH: accession no. GSE299946 and RNA-seq: accession no. GSE299947).

## Supplemental Material

Supplement 1

Supplement 2

Supplement 3
