# Supplementary material for: Plagl1 and Lrrc58 control mammalian body size by triggering target-directed microRNA degradation of miR-322 and miR-503
Source: Genes Dev. 2026 Feb 1;40(3-4):215–32. doi: 10.1101/gad.353138.125 (PMC12863257; doi:10.1101/gad.353138.125)
Supplement: Supplement 3 [file Supplemental_Figures.pdf]

A

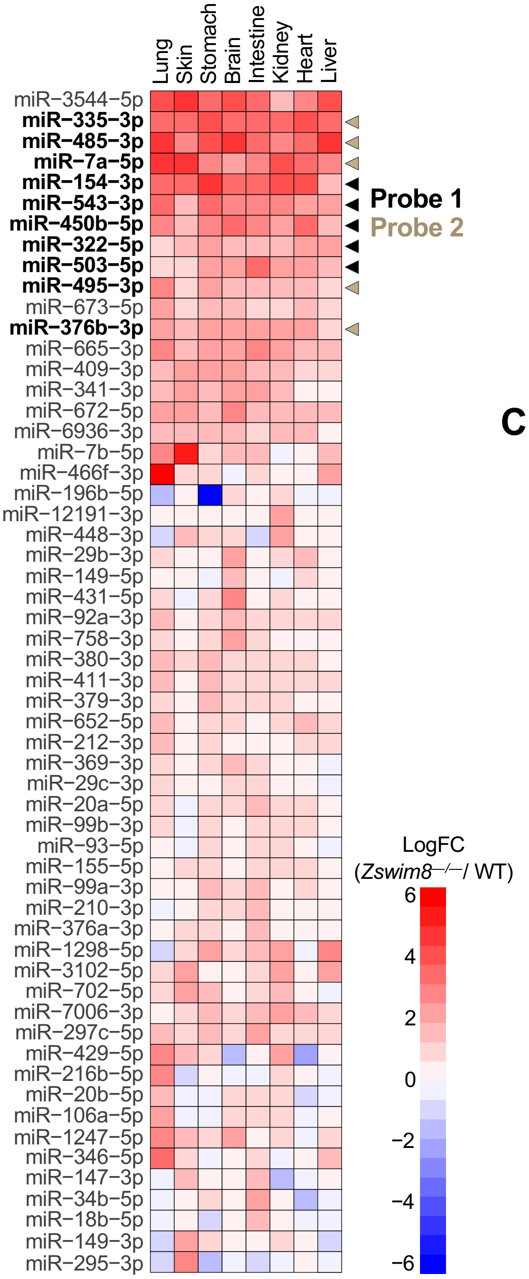

B

	Fraction of chimeric reads		Fraction of chimeras with seed	
	**Rep 1**	**Rep 2**	**Rep 1**	**Rep 2**
WT Brain Probe 1	0.004	0.005	0.087	0.097
Zswim8 $^{-/-}$ Brain Probe 1	0.014	0.016	0.411	0.346
WT Brain Probe 2	0.014	0.009	0.053	0.049
Zswim8 $^{-/-}$ Brain Probe 2	0.078	0.061	0.269	0.243
WT Lung Probe 2	0.012	0.017	0.145	0.143
Zswim8 $^{-/-}$ Lung Probe 2	0.064	0.086	0.343	0.316

C

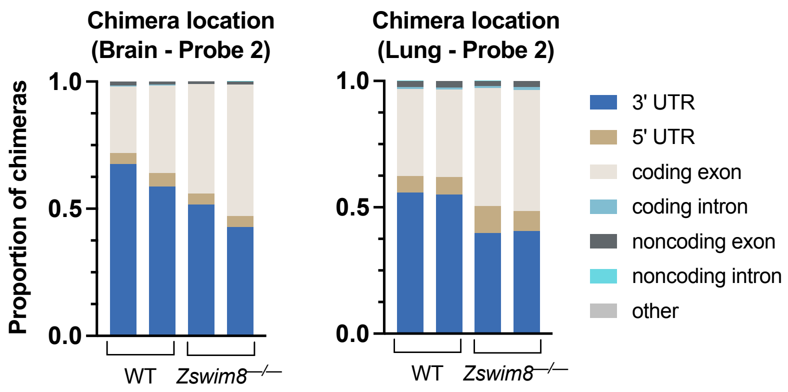

Supplemental Figure 1. miRNA expression in *Zswim8*$^{-/-}$ mice and analysis of AGO-CLASH data, related to Figure 1. (*A*) Heat map showing previously reported $\log_2$ fold change (LogFC) of miRNA expression in E18.5 *Zswim8*$^{-/-}$ versus WT tissues (Jones et al. 2023). miRNAs that were enriched with probes for AGO-CLASH experiments are indicated with arrowheads. (*B*) Table showing the fraction of chimeric reads in AGO-CLASH experiments and the fraction of chimeras comprising a miRNA and a target with a corresponding 6mer, 7mer-A1, 7mer-m8, or 8mer seed match. Rep, replicate. (*C*) Proportion of chimeras mapped to each location in replicate WT and *Zswim8*$^{-/-}$ brain (left) and lung (right) AGO-CLASH samples enriched with Probe 2. Only chimeras in which the predicted base pairing to the miRNA seed sequence was a 6mer, 7mer-A1, 7mer-m8, or 8mer were included in the analysis.

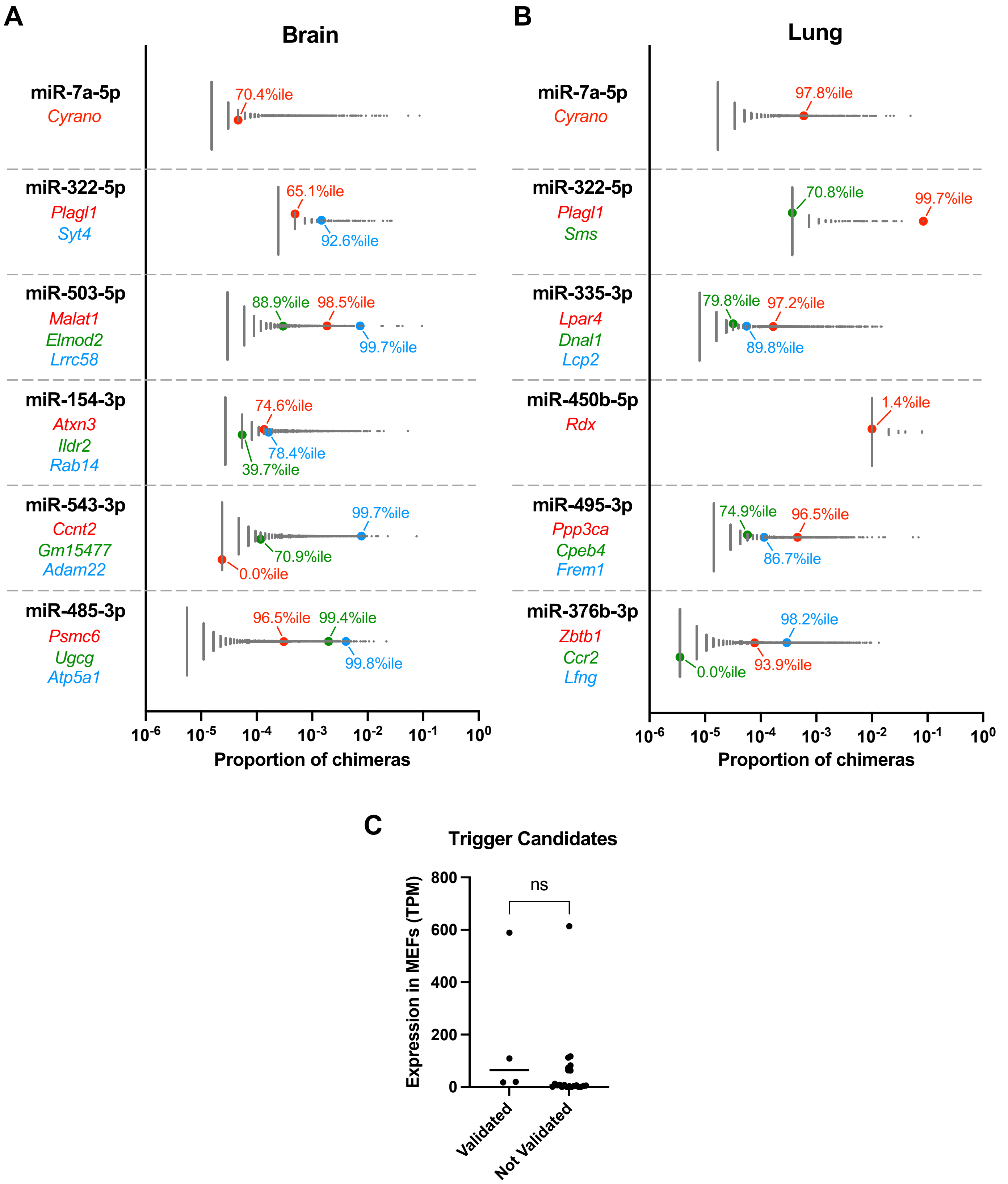

Supplemental Figure 2. Abundance of candidate TDMD trigger RNAs, related to Figure 1. (*A*,*B*) Proportion of chimeras corresponding to each enriched miRNA in AGO-CLASH data from *Zswim8*$^{-/-}$ brain (A) and lung (B). Candidate triggers tested in validation experiments are shown in red (rank 1), green (rank 2), and blue (rank 3), with percentile of abundance for corresponding chimeras indicated on each graph. (*C*) Expression of validated TDMD triggers (*Cyrano, Plagl1, Lrrc58,* and *Malat1*) compared to all other tested candidate triggers in MEFs. TPM, transcripts per million. *P* value was calculated by one-tailed student's t-test with Welch's correction. ns, not significant.

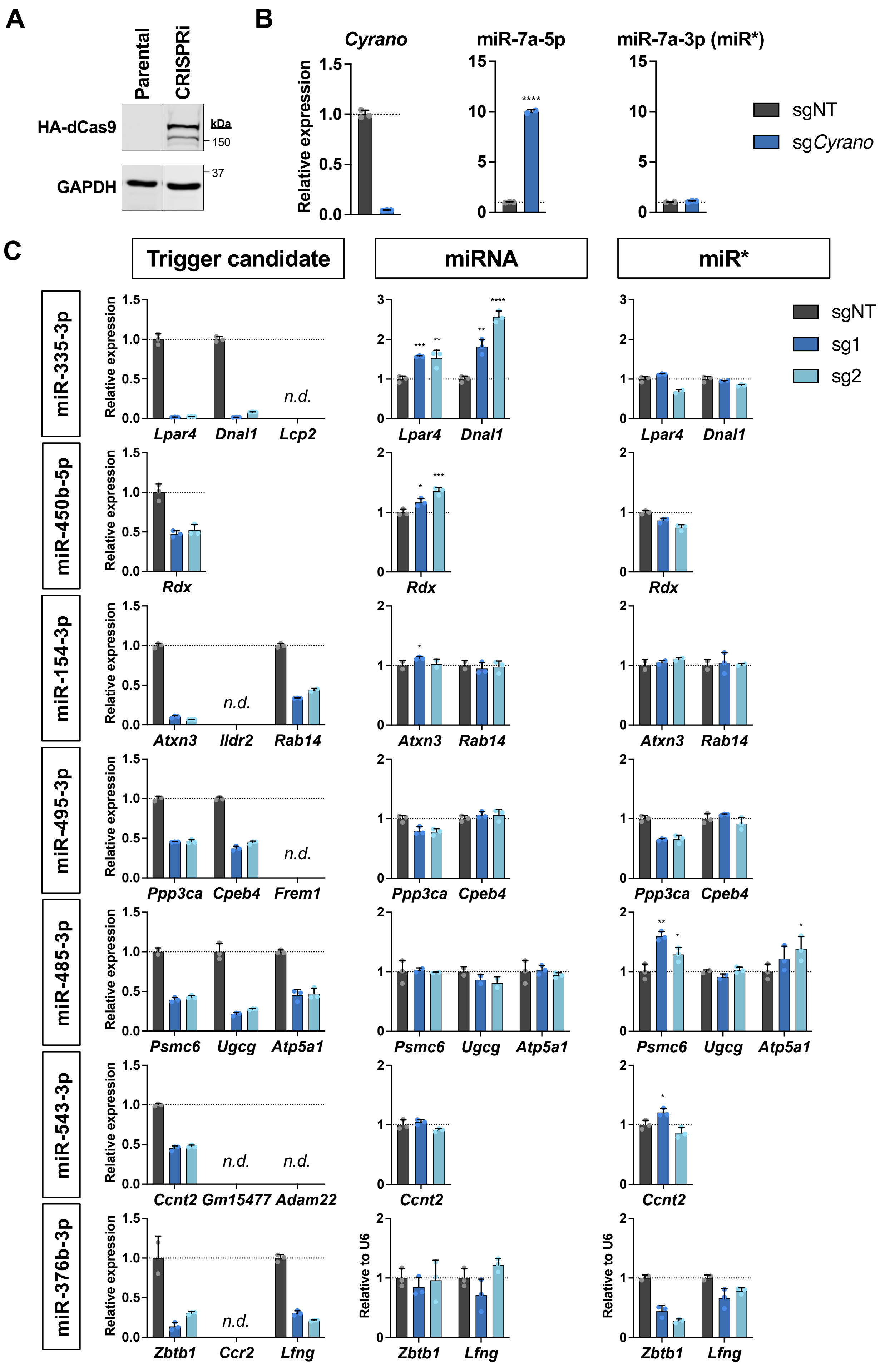
A
Parental
CRISPRi
HA-dCas9
kDa
150
37
GAPDH
B
Cyrano
miR-7a-5p
miR-7a-3p (miR*)
Relative expression
****
sgNT
sgCyrano
C
Trigger candidate
miRNA
miR*
sgNT
sg1
sg2
miR-335-3p
miR-450b-5p
miR-154-3p
miR-495-3p
miR-485-3p
miR-543-3p
miR-376b-3p
Relative expression
Relative to U6
n.d.
Lpar4
Dnal1
Lcp2
Rdx
Atxn3
Ildr2
Rab14
Ppp3ca
Cpeb4
Frem1
Psmc6
Ugcg
Atp5a1
Ccnt2
Gm15477
Adam22
Zbtb1
Ccr2
Lfng

Supplemental Figure 3. CRISPRi-based screening of candidate TDMD trigger RNAs, related to Figure 1. (*A*) Western blot of parental MEFs and a clonal MEF cell line expressing dCas9-HA-BFP-KRAB. GAPDH is shown as loading control. Irrelevant lanes were removed from blots where indicated with vertical lines. (*B*,*C*) dCas9-KRAB-expressing immortalized MEFs were infected with lentivirus encoding a non-targeting guide (sgNT), a guide targeting *Cyrano* (B), or two independent guides targeting each indicated candidate trigger (sg1 or sg2; panel C). Candidate trigger expression was normalized to *Actb* (left), mature miRNA abundance was normalized to miR-16-5p (middle), and passenger strand (miR*) levels were normalized to miR-16-5p (right) except for miR-376b-3p and miR-376b-5p, which were normalized to U6. Values were normalized to expression level in sgNT for each transcript. n=3 technical replicates per sgRNA with individual data points plotted (mean ± SD shown). *P* values were calculated by one-tailed student's t-test comparing sg1 or sg2 to sgNT. *$P<0.05$; **$P<0.01$; ***$P<0.001$; ****$P<0.0001$; n.d., not reliably detected.

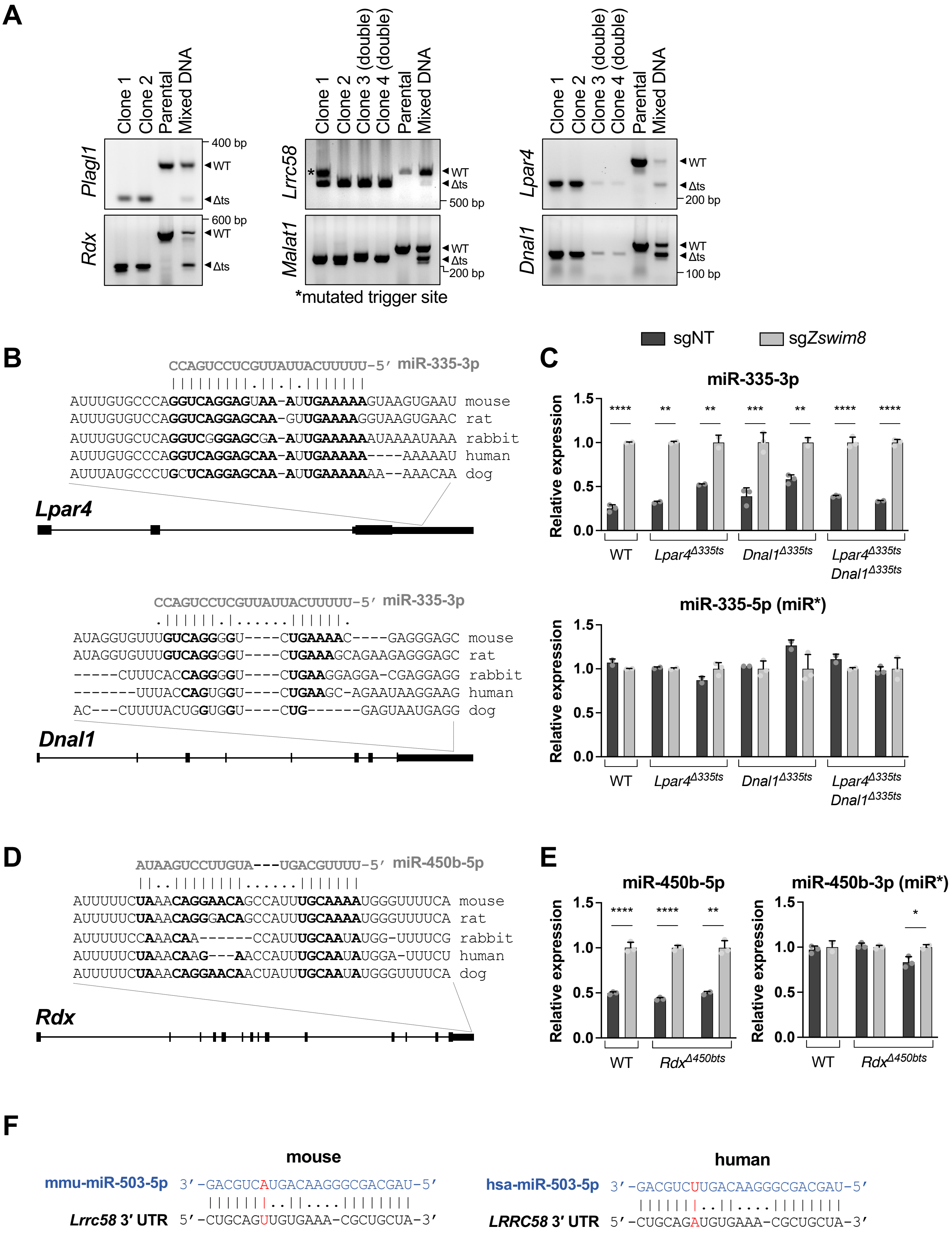
A
Clone 1
Clone 2
Parental
Mixed DNA
Plagl1
Rdx
400 bp
WT
Δts
600 bp
WT
Δts
Clone 1
Clone 2
Clone 3 (double)
Clone 4 (double)
Parental
Mixed DNA
Lrrc58
Malat1
*
WT
Δts
500 bp
WT
Δts
200 bp
*mutated trigger site
Clone 1
Clone 2
Clone 3 (double)
Clone 4 (double)
Parental
Mixed DNA
Lpar4
Dnal1
WT
Δts
200 bp
WT
Δts
100 bp
sgNT
sgZswim8
B
CCAGUCCUCGUUAUUACUUUUU-5′ miR-335-3p
AUUUGUGCCCAGGUCAGGAGUAA-AUUGAAAAAGUAAGUGAAU mouse
AUUUGUGUCCAGGUCAGGAGCAA-GUUGAAAAGGUAAGUGAAC rat
AUUUGUGCUCAGGUCGGGAGCGA-AUUGAAAAAAUAAAAUAAA rabbit
AUUUGUGCCCAGGUCAGGAGCAA-AUUGAAAAA----AAAAAU human
AUUUAUGCCCUGCUCAGGAGCAA-AUUGAAAAAAA--AAACAA dog
Lpar4
CCAGUCCUCGUUAUUACUUUUU-5′ miR-335-3p
AUAGGUGUUUGUCAGGGGU----CUGAAAAC----GAGGGAGC mouse
AUAGGUGUUUGUCAGGGGU----CUGAAAGCAGAAGAGGGAGC rat
-----CUUUCACCAGGGGU----CUGAAGGAGGA-CGAGGAGG rabbit
-------UUUACCAGUGGU----CUGAAGC-AGAAUAAGGAAG human
AC---CUUUUACUGGUGGU----CUG------GAGUAAUGAGG dog
Dnal1
C
miR-335-3p
miR-335-5p (miR*)
Relative expression
1.5
1.0
0.5
0.0
****
**
**
***
**
****
****
WT
Lpar4^Δ335ts
Dnal1^Δ335ts
Lpar4^Δ335ts Dnal1^Δ335ts
D
AUAAGUCCUUGUA---UGACGUUUU-5′ miR-450b-5p
AUUUUUCUAAACAGGAACAGCCAUUUGCAAAAUGGGUUUUCA mouse
AUUUUUCUAAACAGGGACAGCCAUUUGCAAAAUGGGUUUUCA rat
AUUUUUCCAAACAA------CCAUUUGCAAUAUGG-UUUUCG rabbit
AUUUUUCUAAACAAG---AACCAUUUGCAAUAUGGA-UUUCU human
AUUUUUCUAAACAGGAACAACUAUUUGCAAUAUGGGUUUUCA dog
Rdx
E
miR-450b-5p
miR-450b-3p (miR*)
Relative expression
****
****
**
*
WT
Rdx^Δ450bts
F
mouse
human
mmu-miR-503-5p 3′-GACGUCAUGACAAGGGCGACGAU-5′
Lrrc58 3′ UTR 5′-CUGCAGUUGUGAAA-CGCUGCUA-3′
hsa-miR-503-5p 3′-GACGUCUUGACAAGGGCGACGAU-5′
LRRC58 3′ UTR 5′-CUGCAGAUGUGAAA-CGCUGCUA-3′

Supplemental Figure 4. Deletion of additional candidate TDMD trigger sites, related to Figure 2. (*A*) Genotyping PCRs for Δts MEF clones. Genotyping assays were performed on each clone, parental MEFs, and a control sample with mixed WT and Δts DNA. All genotypes were confirmed by nanopore sequencing. One allele in *Lrrc58*$^{\Delta 503ts}$ clone 1 had a mutated trigger site (indicated with asterisk). (*B*,*D*) Genomic organization of candidate TDMD trigger transcripts with conservation and predicted miRNA base-pairing architecture of the trigger sites. Nucleotides predicted to base pair with the miRNA are shown in bold. (*C*,*E*) qRT-PCR analysis of indicated miRNAs, normalized to miR-16-5p, in WT and Δts MEFs. Parental MEFs or two independent Δts clones for each candidate trigger site were infected with lentivirus expressing Cas9 and a non-targeting CRISPR guide (sgNT) or *Zswim8* targeting guide (sg*Zswim8*). Values were normalized to expression level in sg*Zswim8* for each condition. n=3 technical replicates per clone with individual data points plotted (mean ± SD shown). *P* values were calculated by one-tailed student's t-test. *$P<0.05$; **$P<0.01$; ***$P<0.001$; ****$P<0.0001$. (*F*) Predicted base pairing of miR-503-5p to the TDMD trigger site in mouse *Lrrc58* (left) and in human *LRRC58* (right). Nucleotides that differ between mouse and human are shown in red.

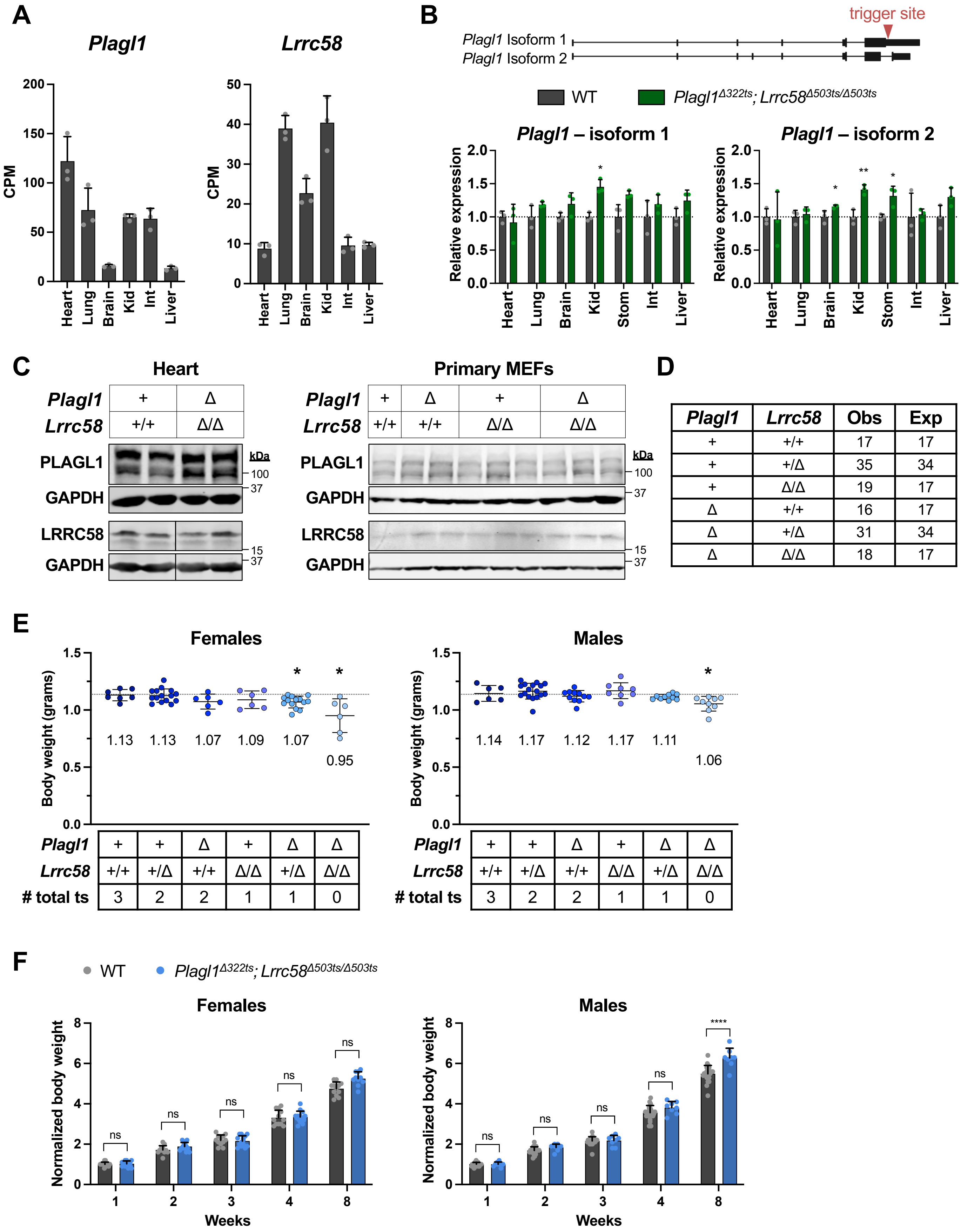

Plagl1	Lrrc58	Obs	Exp
+	+/+	17	17
+	+/Δ	35	34
+	Δ/Δ	19	17
Δ	+/+	16	17
Δ	+/Δ	31	34
Δ	Δ/Δ	18	17

Supplemental Figure 5. ***Plagl1***$^{\Delta 322ts}$**;** ***Lrrc58***$^{\Delta 503ts/\Delta 503ts}$ **mice exhibit reduced body size but normal post-natal growth rate, related to Figure 3.** (*A*) Expression in counts per million (CPM) of *Plagl1* and *Lrrc58* in WT mouse tissues at E18.5, measured by RNA-seq. (*B*) qRT-PCR analysis of alternatively-spliced *Plagl1* isoforms in mouse tissues of the indicated genotypes, normalized to the geometric mean of two housekeeping genes (*Psmd4* and *Oaz1*). Expression was normalized to mean expression in WT in each tissue. Isoform 1 includes the miR-322-5p trigger site in the 3′ UTR, which is spliced out in isoform 2. n=3 mice per genotype, with each mouse represented by an individual data point (mean ± SD shown). *P* values were calculated by one-tailed student's t-test comparing *Plagl1*$^{\Delta 322ts}$*; Lrrc58*$^{\Delta 503ts/\Delta 503ts}$ to WT for each tissue. (*C*) Western blot analysis of PLAGL1 and LRRC58 in E18.5 hearts and in primary MEFs from embryos of the indicated genotypes. GAPDH is shown as a loading control. Irrelevant lanes were removed from blots where indicated with vertical lines. (*D*) Table showing numbers of observed (Obs) and expected (Exp) mice of the indicated genotypes resulting from a *Plagl1*$^{+/\Delta 322ts}$*; Lrrc58*$^{+/\Delta 503ts}$ intercross. Chi-squared=0.647, df=5; two-tailed *P* value=0.995. (*E*) Body weights of female (left) and male (right) E18.5 embryos of the indicated genotypes with each data point representing an individual mouse (mean ±SD shown). Mean weight is denoted on the graph below each cohort. Dotted line is the mean weight of the WT cohort. n=6-17 mice per genotype. *P* values were calculated by one-tailed student's t-test comparing each genotype to WT. ts, trigger site. (*F*) Graph of body weights of WT and *Plagl1*$^{\Delta 322ts}$; *Lrrc58*$^{\Delta 503ts/\Delta 503ts}$ mice at the indicated timepoints normalized to the average weight of each respective genotype at 1-week of age (mean ±SD shown). n=8-22 mice for each genotype at each timepoint. *P* values were calculated by one-tailed student's t-test. **P*<0.05; ***P*<0.01; *****P*<0.0001; ns, not significant.

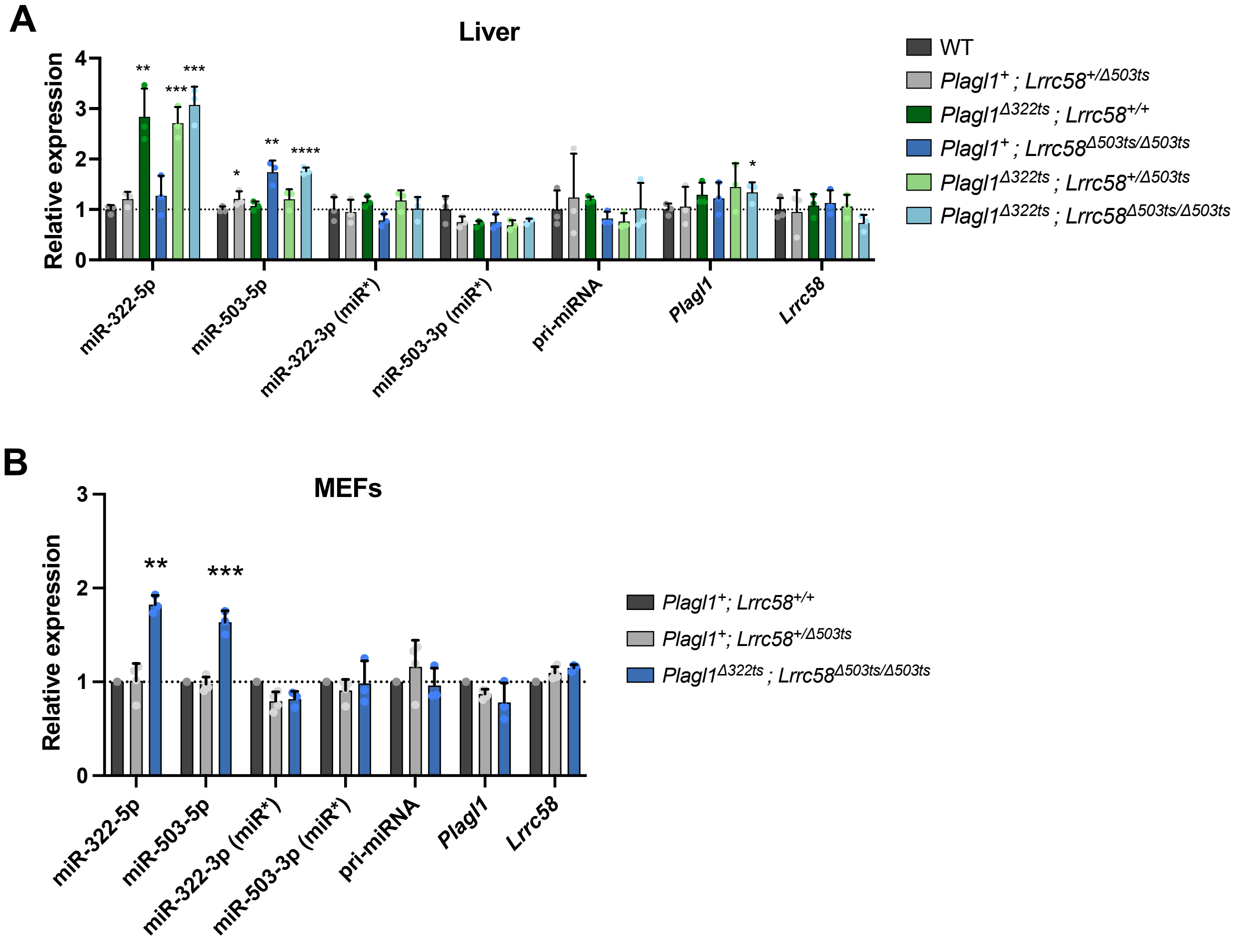

Supplemental Figure 6. Loss of the *Plagl1* and *Lrrc58* trigger sites abrogates TDMD of miR-322-5p and miR-503-5p *in vivo*, related to Figure 4. (*A*) qRT-PCR analysis of the indicated transcripts in E18.5 livers. Expression of each transcript was normalized to mean expression in WT. n=3 biological replicates per genotype with individual data points shown (mean ± SD shown). *P* values were calculated by one-tailed student's t-test comparing each genotype to WT. (*B*) qRT-PCR analysis of the indicated transcripts in primary MEFs. Expression of each transcript was normalized to expression in $Plagl1^{+}$; $Lrrc58^{+/+}$. n=1 biological replicate for $Plagl1^{+}$; $Lrrc58^{+/+}$, n=4 biological replicates for $Plagl1^{+}$; $Lrrc58^{+/\Delta 503ts}$, and n=3 biological replicates for $Plagl1^{\Delta 322ts}$; $Lrrc58^{\Delta 503ts/\Delta 503ts}$ with individual data points shown (mean ± SD shown). *P* values were calculated by one-tailed student's t-test comparing $Plagl1^{\Delta 322ts}$; $Lrrc58^{\Delta 503ts/\Delta 503ts}$ to $Plagl1^{+}$; $Lrrc58^{+/\Delta 503ts}$. *P<0.05; **P<0.01; ***P<0.001; ****P<0.0001.
